# Supplementary material for: Spatial transcriptomic validation of a biomimetic model of fibrosis enables re-evaluation of a therapeutic antibody targeting LOXL2
Source: Cell Rep Med. 2024 Aug 21;5(9):101695. doi: 10.1016/j.xcrm.2024.101695 (PMC11524965; doi:10.1016/j.xcrm.2024.101695)
Supplement: Document S1. Figures S1–S4 and Table S1 [file mmc1.pdf]

Cell Reports Medicine, Volume *5*

## Supplemental information

# Spatial transcriptomic validation of a biomimetic model of fibrosis enables re-evaluation of a therapeutic antibody targeting LOXL2

**Joseph A. Bell, Elizabeth R. Davies, Christopher J. Brereton, Milica Vukmirovic, James J.W. Roberts, Kerry Lunn, Leanne Wickens, Franco Conforti, Robert A. Ridley, Jessica Ceccato, Lucy N. Sayer, David A. Johnston, Andres F. Vallejo, Aiman Alzetani, Sanjay Jogai, Ben G. Marshall, Aurelie Fabre, Luca Richeldi, Phillip D. Monk, Paul Skipp, Naftali Kaminski, Emily Offer, Yihua Wang, Donna E. Davies, and Mark G. Jones**

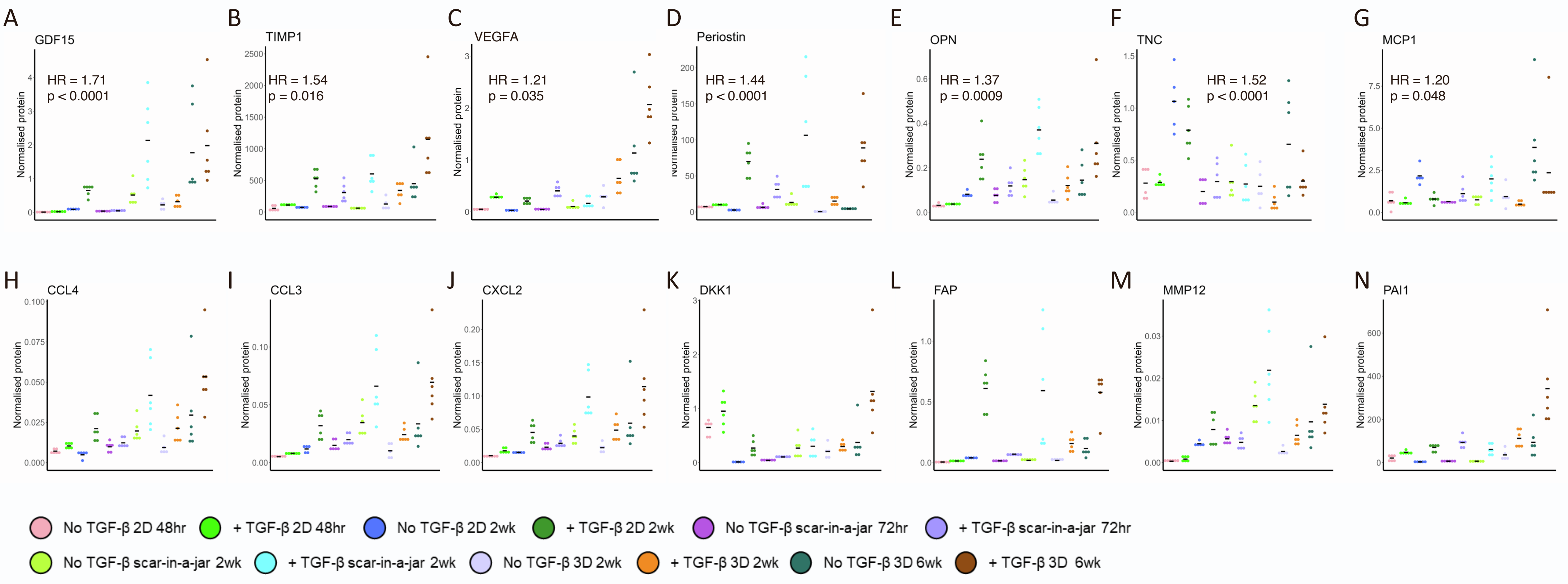


**Figure S1. Cytokine and extracellular protein profiles of cell conditioned media from each model system,** Related to Figure 3. Individual cytokines and extracellular proteins from conditioned media from each model system. A primary parenchymal lung fibroblast cell line was cultured in standard 2D culture, the Scar-in-a-jar model or the 3D spheroid model in the absence or presence of TGF-β at early and late timepoints (n=6 per condition). (A) GFD15, (B) TIMP1, (C) VEGFA (D) Periostin (E) OPN (osteopontin), (F) TNC), (G), MCP1 (CCL2), (H) CCL4, (I) CCL3, (J) CXCL2, (K) DKK1, (L) FAP, (M) MMP12, (N) PAI1 (A-G) proteins which have a significant hazard ratio > 1 for increased risk of death or transplant in Oldham et al. 2023[58]. Hazard ratios and P values from this study are inset in graphs. Supernatants derived from each model system were taken and analyte concentration in the supernatant was quantified against a standard curve using a Human Luminex Discovery Assay Kit analysed on the Magpix Luminex platform. Luminex data were normalised to total RNA and media volume to allow comparison across different culture models.

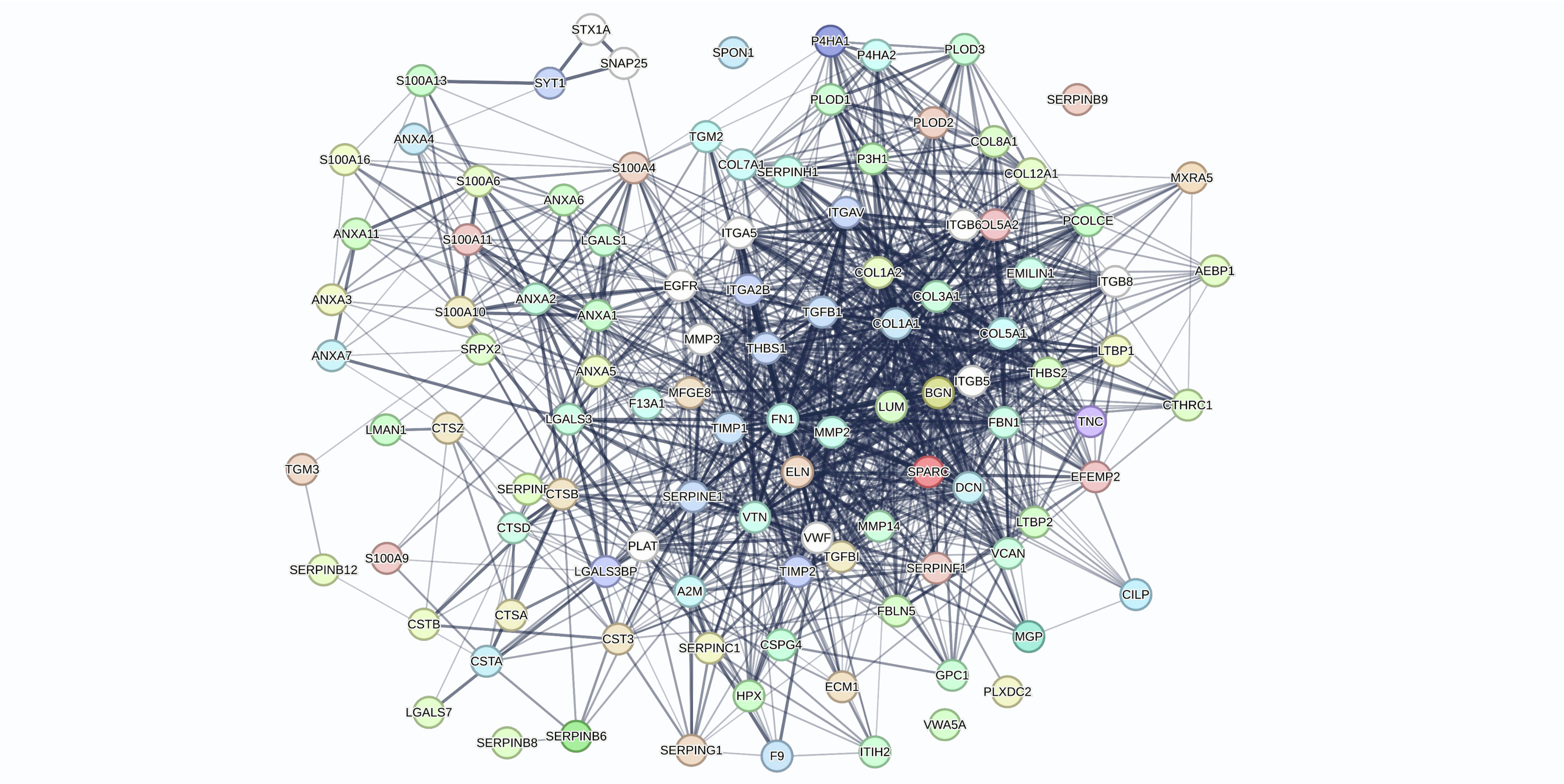


**Figure S2. Interaction network map of common proteins between 3D spheroid model and fibroblastic foci,** Related to Figure 3. STRING map with up to 10 additional medium-confidence interactors for 88 common extracellular matrix proteins between IPF fibroblastic foci and 3D spheroid model (see Fig. 4a and Supplementary Table 2). Thickness of lines denotes confidence of interaction.

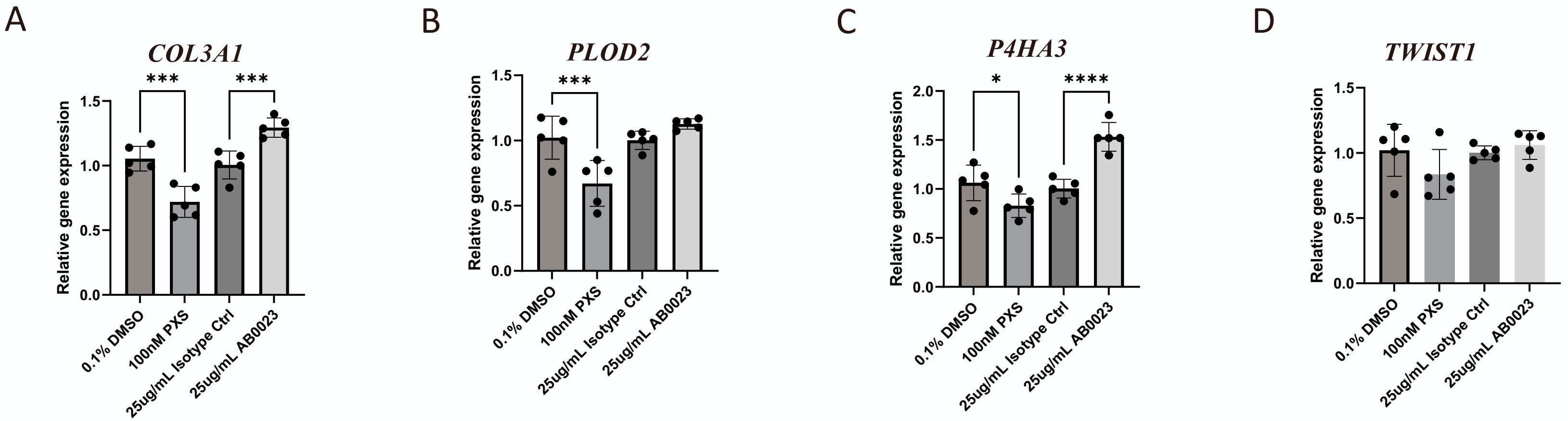


**Figure S3. PXS-S2A decreases expression of profibrotic genes,** Related to Figure 5. Primary human lung fibroblasts were cultured in 3D spheroid media conditions in the presence of TGF-β and treated with AB0023, an isotype control antibody at the same concentration, PXS-S2A or its vehicle control (0.1% DMSO). Relative gene expression for (A) *COL3A1* (B) *PLOD2* (C) *P4HA3* (D) *TWIST1* determined by qRT-PCR and analyzed using DDCt method (n=5 replicates per condition across 2 independent experiments). *P<0.05, **P<0.01, ***P<0.001, ****P<0.0001. One-way ANOVA with Šídák's multiple comparisons test was used to evaluate statistical significance. (F values: (A) 27.33, (B) 12.12, (C) 23.16, Degrees of Freedom: 16.

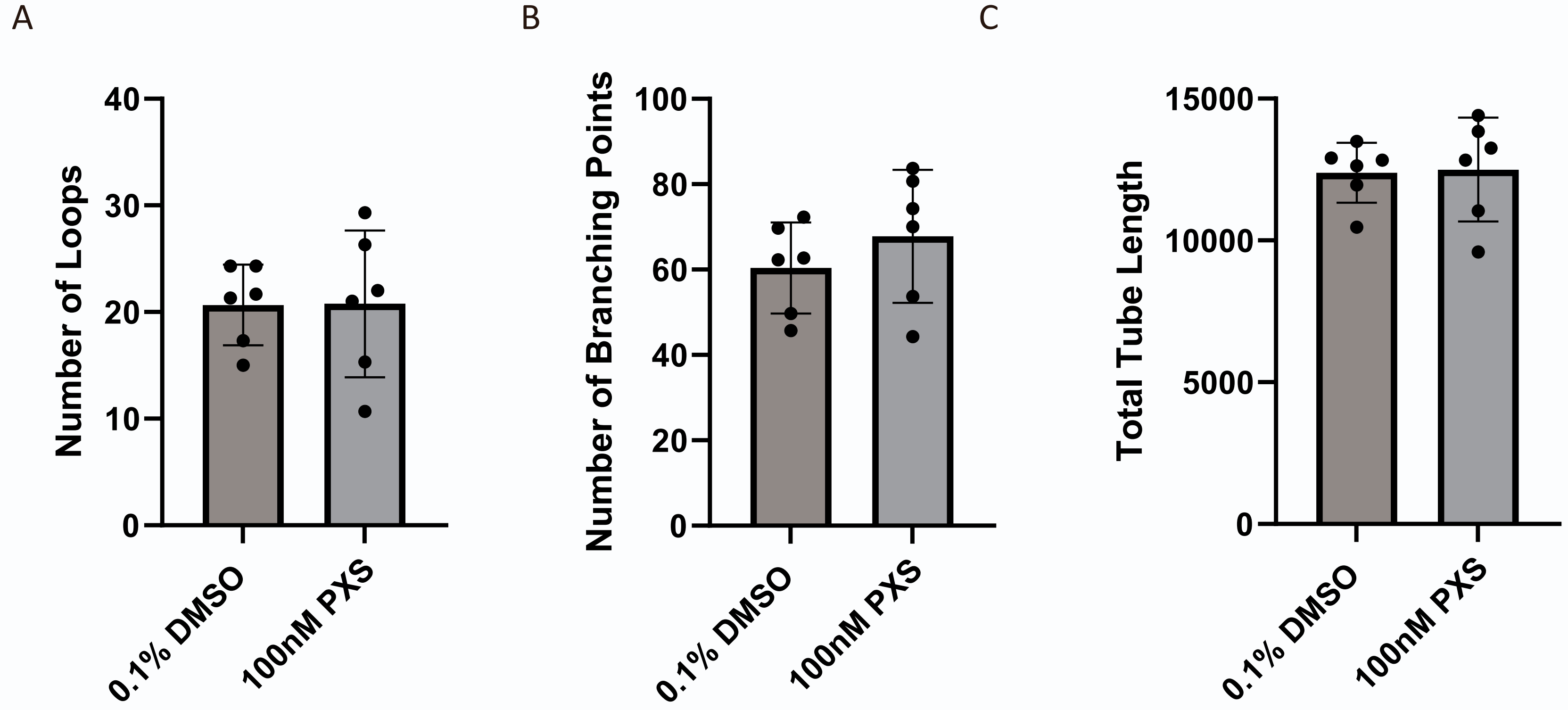


**Figure S4. Selective targeting of LOXL2 catalytic activity using the small molecule inhibitor PXS-S2A does not inhibit endothelial tube formation,** Related to Figure 6. Endothelial tube formation by human umbilical vein endothelial cells was assessed in the presence of PXS-S2A or 0.1% DMSO (n=6 replicates per condition across 2 independent experiments). Quantification of topological parameters of capillary structure by computer-aided image analysis for (A) number of loops, (B) number of branching points, (C) total tube length. Data are means +/- SD.

| Sex | Age | Disease status |
|---|---|---|
| M | 67 | IPF |
| M | 66 | IPF |
| F | 63 | IPF |
| M | 69 | IPF |
| F | 74 | IPF |
| M | 69 | IPF |
| F | 75 | IPF |
| M | 73 | IPF |
| M | 71 | IPF |
| M | 73 | IPF |
| F | 73 | Normal |
| M | 68 | Normal |
| M | 64 | Normal |
| M | 76 | Normal |
| F | 61 | Normal |
| M | 75 | Normal |
| M | 70 | Normal |
| M | 72 | Normal |
| F | 67 | Normal |
| M | 63 | Normal |

**Table S1. Demographic details of donors,** related to Figures 1-5.

**Supplemental References**


S1. Herrera, J.A., Dingle, L., Montero, M.A., Venkateswaran, R.V., Blaikley, J.F., Lawless, C., and Schwartz, M.A. (2022). The UIP/IPF fibroblastic focus is a collagen biosynthesis factory embedded in a distinct extracellular matrix. JCI Insight 7, e156115.

S2. Naba, A., Clauser, K.R., Ding, H., Whittaker, C.A., Carr, S.A., and Hynes, R.O. (2016). The extracellular matrix: Tools and insights for the “omics” era. Matrix Biol. 49, 10–24.

S3. Oldham, J.M., Huang, Y., Bose, S., Ma, S.-F., Kim, J.S., Schwab, A., Ting, C., Mou, K., Lee, C.T., Adegunsoye, A., et al. (2024). Proteomic Biomarkers of Survival in Idiopathic Pulmonary Fibrosis. Am. J. Respir. Crit. Care Med. 209, 1111-1120.
