# Supplementary material for: Spatial transcriptomic validation of a biomimetic model of fibrosis enables re-evaluation of a therapeutic antibody targeting LOXL2
Source: Cell Rep Med. 2024 Aug 21;5(9):101695. doi: 10.1016/j.xcrm.2024.101695 (PMC11524965; doi:10.1016/j.xcrm.2024.101695)
Supplement: Document S2. Article plus supplemental information [file mmc5.pdf]

Cell Reports
Medicine

Article

Spatial transcriptomic validation of a biomimetic model of fibrosis enables re-evaluation of a therapeutic antibody targeting LOXL2

Graphical abstract

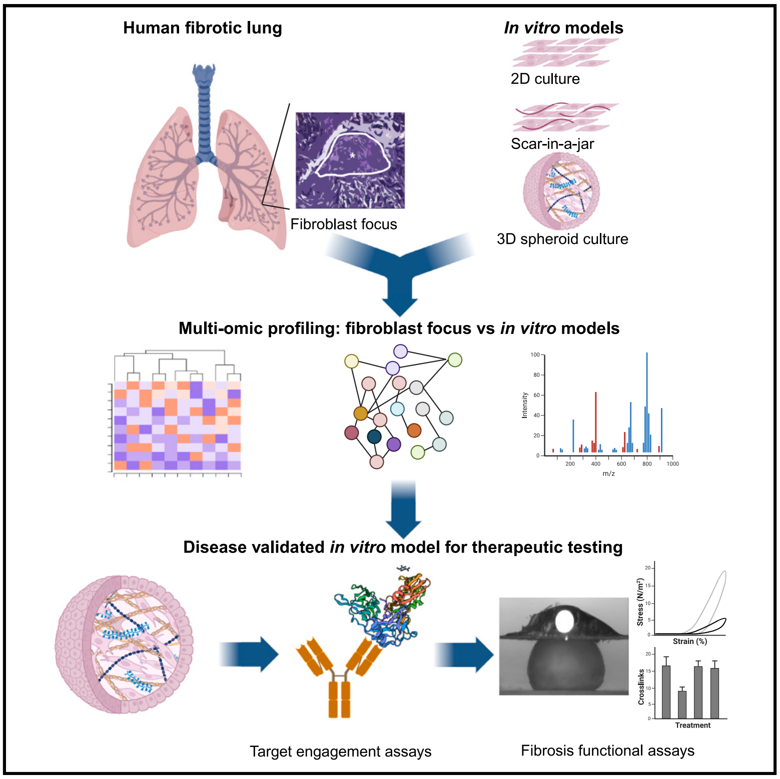

Highlights

- Spatial multi-omics identifies a disease-relevant model of lung fibrosis
- A 3D model recapitulates gene and protein expression patterns of fibroblast foci
- Target engagement and functional fibrosis assays within the validated 3D model
- Anti-LOXL2 antibody AB0023/simtuzumab does not inhibit LOXL2 catalytic activity

Authors

Joseph A. Bell, Elizabeth R. Davies, Christopher J. Brereton, ..., Yihua Wang, Donna E. Davies, Mark G. Jones

Correspondence

mark.jones@soton.ac.uk

In brief

Bell et al. compare the spatial transcriptome of human lung fibrogenesis with cell culture-based models. A 3D model recapitulates the gene expression profile of active fibrogenesis sites. Applying the model to investigate an antibody proposed to target LOXL2 that failed in clinical trials, the antibody does not inhibit LOXL2 activity.

Bell et al., 2024, Cell Reports Medicine *5*, 101695
September 17, 2024 © 2024 The Author(s). Published by Elsevier Inc.
https://doi.org/10.1016/j.xcrm.2024.101695

CellPress

Cell Reports Medicine

CellPress
OPEN ACCESS

Article

Spatial transcriptomic validation of a biomimetic model of fibrosis enables re-evaluation of a therapeutic antibody targeting LOXL2

Joseph A. Bell,[1,2] Elizabeth R. Davies,[1,2,3] Christopher J. Brereton,[1,2] Milica Vukmirovic,[4,5] James J.W. Roberts,[6] Kerry Lunn,[6] Leanne Wickens,[1,2,7] Franco Conforti,[1,2] Robert A. Ridley,[1,2] Jessica Ceccato,[1,8] Lucy N. Sayer,[1,2] David A. Johnston,[9] Andres F. Vallejo,[1] Aiman Alzetani,[2,10] Sanjay Jogai,[10] Ben G. Marshall,[2,10] Aurelie Fabre,[11] Luca Richeldi,[1,2,12] Phillip D. Monk,[6] Paul Skipp,[2,3,10] Naftali Kaminski,[4] Emily Offer,[13] Yihua Wang,[2,3,7] Donna E. Davies,[1,2,7] and Mark G. Jones[1,2,7,14,*]

[1]Clinical and Experimental Sciences, Faculty of Medicine, University of Southampton, SO16 6YD Southampton, UK
[2]NIHR Southampton Biomedical Research Centre, University Hospital Southampton, SO16 6YD Southampton, UK
[3]Biological Sciences, Faculty of Environmental and Life Sciences, University of Southampton, SO17 1BJ Southampton, UK
[4]Section of Pulmonary, Critical Care and Sleep Medicine, Department of Medicine, Yale University School of Medicine, New Haven, CT, USA
[5]Leslie Dan Faculty of Pharmacy, University of Toronto, Toronto, ON, Canada
[6]Synairgen Research Ltd, Southampton, UK
[7]Institute for Life Sciences, University of Southampton, SO17 1BJ Southampton, UK
[8]Department of Medicine, University of Padova, Padova, Italy
[9]Biomedical Imaging Unit, Faculty of Medicine, University of Southampton, Southampton, UK
[10]University Hospital Southampton, SO16 6YD Southampton, UK
[11]Department of Histopathology, St. Vincent's University Hospital & UCD School of Medicine, University College Dublin, Dublin, Ireland
[12]Unità Operativa Complessa di Pneumologia, Università Cattolica del Sacro Cuore, Fondazione Policlinico A. Gemelli, Rome, Italy
[13]Medicines Discovery Catapult, Alderley Edge, UK
[14]Lead contact
*Correspondence: mark.jones@soton.ac.uk
https://doi.org/10.1016/j.xcrm.2024.101695

SUMMARY

Matrix stiffening by lysyl oxidase-like 2 (LOXL2)-mediated collagen cross-linking is proposed as a core feed-forward mechanism that promotes fibrogenesis. Failure in clinical trials of simtuzumab (the humanized version of AB0023, a monoclonal antibody against human LOXL2) suggested that targeting LOXL2 may not have disease relevance; however, target engagement was not directly evaluated. We compare the spatial transcriptome of active human lung fibrogenesis sites with different human cell culture models to identify a disease-relevant model. Within the selected model, we then evaluate AB0023, identifying that it does not inhibit collagen cross-linking or reduce tissue stiffness, nor does it inhibit LOXL2 catalytic activity. In contrast, it does potently inhibit angiogenesis consistent with an alternative, non-enzymatic mechanism of action. Thus, AB0023 is anti-angiogenic but does not inhibit LOXL2 catalytic activity, collagen cross-linking, or tissue stiffening. These findings have implications for the interpretation of the lack of efficacy of simtuzumab in clinical trials of fibrotic diseases.

INTRODUCTION

Fibrotic diseases are a major cause of morbidity and mortality in the developing world. Within the lung, idiopathic pulmonary fibrosis (IPF) is considered the prototypic chronic progressive fibrotic disease.[1] Treatment options are limited, and with a median survival of less than 3 years from diagnosis, more effective therapies are urgently needed. While the exact mechanisms are uncertain, progressive lung fibrosis is believed to result from repetitive micro-injuries to the alveolar epithelium promoting aberrant fibroblast activation into matrix-producing myofibroblasts.[1] These myofibroblasts deposit extracellular matrix (ECM) components, which eventually destroy normal alveolar architecture with a consequent disruption of gas exchange. Pathogenic ECM changes have been strongly implicated in fibrosis progression, with resulting increased matrix stiffness proposed to induce persistent mesenchymal cell activation and hence fibrosis progression in a positive feedback loop.[2–7]

A defining feature of human idiopathic IPF (usual interstitial pneumonia pattern) is spatial heterogeneity, with normal lung adjacent to histologically evident established fibrosis. At the interface between fibrosis and morphologically normal lung are fibroblast foci; our own studies and those of others evidence that they reflect discrete sites of active fibrogenesis.[8–10] Importantly, the density of fibroblast foci is the histologic feature

© 2024 The Author(s). Published by Elsevier Inc.
This is an open access article under the CC BY license (http://creativecommons.org/licenses/by/4.0/).

most consistently associated with disease progression,[11–13] consistent with their having a key pathogenetic role in lung fibrosis, with histological studies identifying them to be mesenchymal cells that are synthesizing altered ECM. Thus, increased understanding of fibroblast foci and their *in vitro* recapitulation provides the opportunity to dissect key aspect(s) of progressive human lung fibrogenesis.

Fibrillar collagens are a major component of ECM that form a scaffold to support tissue architecture and are a primary determinant of tissue stiffness in health and disease.[14,15] The tensile properties of collagen fibrils result from intermolecular cross-links connecting the nonhelical ends of a collagen molecule (telopeptides) with the triple helical part of an adjacent molecule.[16,17] The lysyl oxidase (LOX) enzymes are a family of five secreted copper-dependent amine oxidases that are responsible for post-translational modification of collagen in the ECM by initiating this covalent cross-linking process. LOX enzymes convert specific lysine or hydroxylysine residues in the telopeptides into the aldehydes allysine and hydroxyallysine, respectively.[18] The aldehydes subsequently react with lysine, hydroxylysine or histidyl, and residues of the triple helix to give characteristic di-, tri-, and tetrafunctional cross-links. Cross-linking is essential to stabilize the supramolecular assembly of collagen and produce stable collagen fibrils. Dysregulation of LOX family member expression has been identified across many disease areas,[19] with increased mature trivalent pyridinoline (PYD) and deoxypyridinloine (DPD) hydroxyallysine-derived collagen cross-links altering collagen nano-architecture and increasing tissue stiffness in human lung fibrosis.[15]

LOX-like 2 (LOXL2)-mediated collagen cross-linking has been proposed as a core pathway of fibrogenesis in multiple fibrotic diseases including the lung, heart, and liver.[20,21] Within the lung, LOXL2 is highly expressed within fibroblastic foci, the sites of active fibrogenesis,[22] while elevated LOXL2 levels in serum have been associated with increased risk for IPF disease progression in two cohorts of patients.[23] AB0023, a monoclonal antibody against human LOXL2 protein, showed efficacy in the bleomycin mouse model of lung fibrosis as well as in preclinical models of liver fibrosis and cardiac fibrosis.[20,21] However, simtuzumab (the fully humanized version of AB0023) failed to achieve positive clinical endpoints in multiple fibrotic diseases including IPF.[24–26] Importantly, no direct evidence of target engagement in these studies was measured, triggering uncertainty about the reason for the lack of efficacy in humans. Furthermore, while AB0023 was proposed in preclinical studies to partially inhibit LOXL2-mediated enzymatic activity, this was based on the measurement of amine oxidase activity in cell-free biochemical assays[27]; however, no direct biochemical measurement of inhibition of collagen cross-linking was performed.[20,27] Thus, in the absence of direct assessment of molecular mechanism of action (i.e., inhibition of collagen cross-linking activity), the impact of simtuzumab/AB0023 on pathological tissue stiffening is still unknown.

Multiple animal models of lung fibrosis have been developed; however, significant numbers of proposed therapies with demonstrated efficacy in animal studies have translated into ineffective or even harmful actions in clinical studies.[28–30] Importantly, the most commonly used murine model of bleomycin-induced lung fibrosis does not recapitulate key features of human lung fibrogenesis including increased mature PYD cross-linking[31] and so cannot be used to dissect key factors promoting altered collagen cross-linking in human disease. To reduce the attrition rate of new drugs, greater emphasis is therefore being placed on *in vitro* human cell-based biomimetic models, which may better recapitulate human disease.[32] For cellular models of fibrosis, matrix-producing fibroblasts are typically cultured as monolayers on standard tissue culture plastic; however, substrate stiffness, composition, and structure are known to influence cell behavior and phenotype.[33] Advanced 3D cell culture methodologies using disease-relevant cells have therefore been proposed to better reproduce the complex cell-cell and cell-ECM environment of human lung fibrosis,[34] but only limited validation of the disease relevance of such approaches has been performed.

Recent advances in RNA sequencing and spatial transcriptomic approaches provide the opportunity to characterize gene expression profiles within distinct regions of the fibrotic niche including cell populations and key regulatory pathways active within areas of active fibrogenesis.[35,36] The spatial transcriptome of the fibrotic niche can then be compared with *in vitro* culture model systems to identify models with most disease relevance.

Here, we compared the spatial transcriptome of fibroblast foci with cell culture-based models to identify the *in vitro* model that most closely recapitulates the gene expression profile of human fibroblast foci. We then investigated directly the efficacy of AB0023 upon collagen cross-linking and tissue stiffness within this model.

RESULTS

Enrichment of a skeletal development signature within fibroblastic foci

We firstly studied the spatial transcriptomic profiles of fibroblast foci, which are the sites of active fibrogenesis in IPF. We analyzed a dataset we recently generated by integrating laser-capture microdissection and RNA sequencing to profile the *in situ* transcriptome of fibroblast foci as well as alveolar septae from control tissue and IPF tissue.[22] We confirmed biologically plausible clustering of each region of interest by principal component analysis (Figure 1A), with this most apparent for fibroblast foci, which cluster together distant from the alveolar septae samples. We then confirmed enrichment of key mesenchymal-associated genes within the fibroblastic foci including *ACTA2* (Figure 1B) as well as matrisomal genes including *TNC*, *COL1A1*, and *COL5A1* (Figures 1C–1E). In contrast, the type 2 alveolar epithelial gene *SFTPC* was significantly reduced within fibroblast foci (Figure 1F) when compared with alveolar septae.

In order to dissect gene sets enriched in the fibroblast foci, gene set variation analysis (GSVA) was performed. We identified a strong enrichment for Gene Ontology terms associated with collagen fibril organization and bone morphogenesis within fibroblast foci (Figures 1G–1I, and Data S1), a finding in keeping with our recent spatial analysis of gene expression profiles of fibroblast foci through digital spatial profiling.[36]

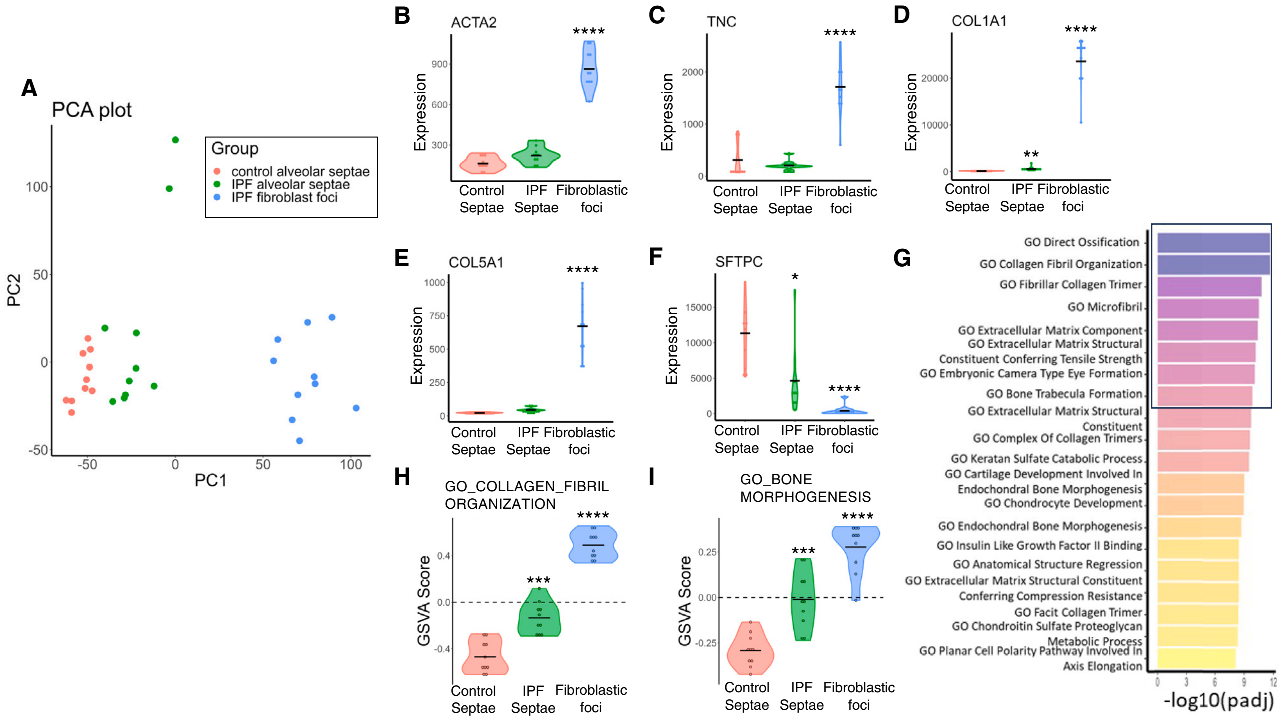

Figure 1. The spatial transcriptome of fibroblast foci is enriched for ECM development and ossification gene expression signatures

(A) Principal component analysis (PCA) plot showing variance of the transcriptome (GSE169500) of laser-capture microdissection (LCMD) samples for control alveolar septae, IPF alveolar septae, and IPF fibroblast foci (n = 10 control and IPF donors).

(B–F) Violin plots showing expression of *ACTA2* (B), *TNC* (C), *COL1A1* (D), *COL5A1* (E), and *SFTPC* (F). Relative expression levels are calculated as counts per million reads (CPM). Fibroblastic foci gene expression values are compared with control alveolar septae. (G) Gene set variation analysis (GSVA) of LCMD data, showing top 20 significantly enriched Gene Ontology (GO) terms in IPF fibroblastic foci compared to control alveolar septae. GO terms are ranked by $-\log_{10}$(adjusted p value).

(H, I) Violin plots showing GSVA scores for GO terms collagen fibril organization (H) and bone morphogenesis (I). p values calculated using the EdgeR R package (B–F) and the Limma R package (H, I) comparing fibroblastic foci and IPF alveolar septae to control septae using Benjamini-Hochberg multiple test correction $^*p < 0.05$, $^{**}p < 0.01$, $^{***}p < 0.001$, $^{****}p < 0.0001$.

A 3D spheroid model most closely resembles the transcriptome of human fibroblast foci

To translate the insights from our clinical sample analysis, we performed cell culture studies. In order to determine a model of fibrogenesis that can best represent human fibroblast foci, we compared the transcriptome of fibroblast foci with 3 primary human lung parenchymal fibroblast cell culture models using their standard culture conditions in the presence or absence of the profibrotic cytokine TGF-β_1: (1) a 2D model of primary lung fibroblasts grown on standard tissue culture plasticware,[37] (2) a pseudo-3D Scar-in-a-Jar model using macromolecules to provide crowding conditions proposed to more closely resemble the fibrotic microenvironment and promote collagen deposition,[38] and (3) a long-term 3D spheroid model that self-assembles a 3D ECM with a progressive increase in collagen content, which we have previously demonstrated enables direct evaluation of PYD cross-linking, collagen nanostructure, and tissue biomechanics.[15] For each culture model, a second time point (2 weeks) was included to allow for overlap of culture duration. An overview of the study design is presented in Figure 2A, and example microscopy images of each model in Figure 2B.

To compare these different culture models to fibroblastic foci, we performed GSVA on a combined dataset of the transcriptome of fibroblastic foci and of each cell culture model. Unsupervised hierarchical clustering of GSVA scores for the most variable Gene Ontology terms (Kruskal-Wallis test, $p < 0.01$) identified that the 3D spheroid model clustered together with fibroblast foci (Figure 2C), with the greatest similarity between fibroblast foci and the 3D spheroid model in the presence of TGF-β_1. This condition exhibited expression patterns involving morphology (GO_Actin_Filament) (Figure 2D), signaling pathways (GO_Chemokine_Activity & GO_Non-canonical_Wnt_Signaling_Pathway) (Figures 2E and 2F), and metabolism (GO_Tricarboxylic_Acid_Cycle) (Figure 2G), as well as pathways associated with bone morphogenesis (GO_Osteoclast_Proliferation) (Figure 2H) similar to those enriched within our spatial transcriptomic analysis of fibroblast foci. In contrast, the Scar-in-a-Jar and 2D culture models clustered together (Figure 2C), with distinct subclusters of each model and time point in the absence or presence of TGF-β_1.

To understand which gene expression patterns within the 3D spheroid model may support the similarity with the fibroblast focus transcriptome, we performed analysis of significantly

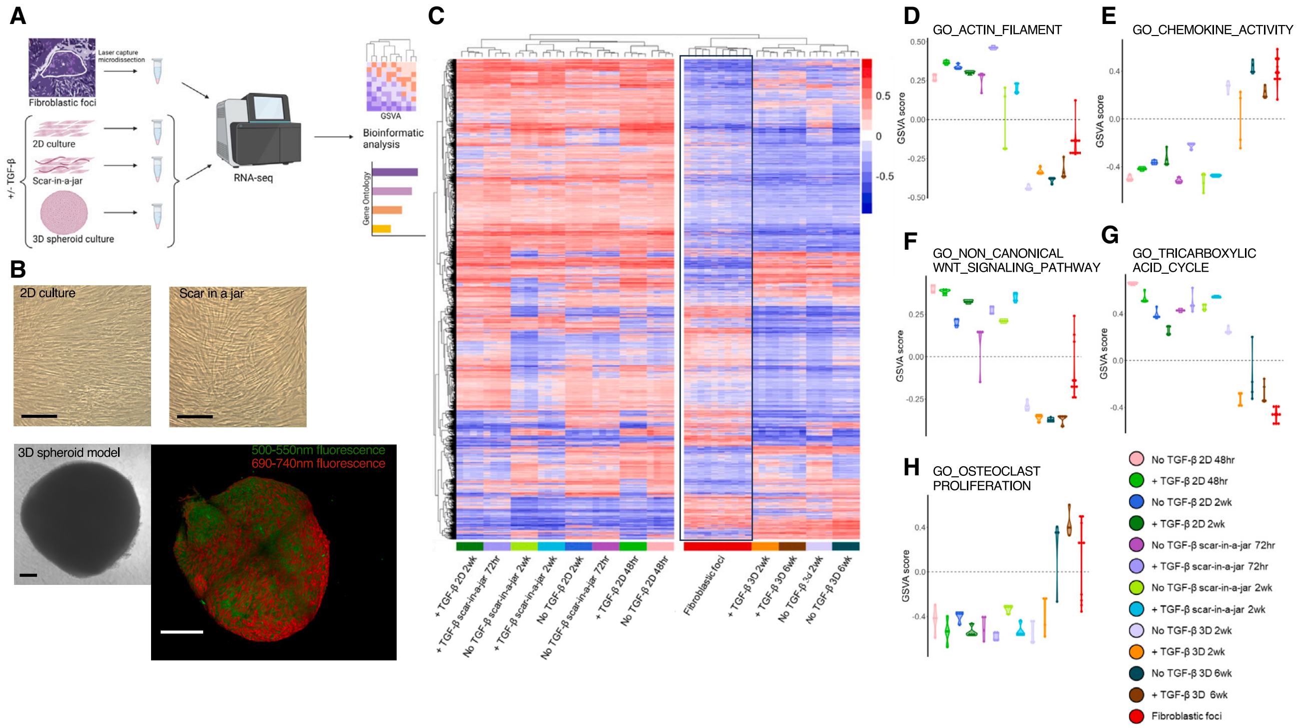

Figure 2. The 3D spheroid model most closely reflects the transcriptome of fibroblast foci

(A) Schematic representation of the experimental design. Primary parenchymal lung fibroblasts were cultured in standard 2D culture, the Scar-in-a-jar model, or the 3D spheroid model in the absence or presence of TGF-β at early and late time points ($n = 4$ per condition) and each transcriptome compared through bioinformatic analysis with the transcriptome of fibroblast foci.

(B) Representative phase-contrast micrographs of standard 2D culture, scar-in-a-jar, and 3D spheroid model. The 3D morphology of spheroids is visualized by confocal microscopy utilizing fluorescence of carmine red staining at bandwidths of 500–550 and 690–740 nm (green and red false colors, respectively). Scale bars are all 200 μm.

(C) Heatmap representing unsupervised hierarchical clustering of highly variable GSVA scores in models and fibroblastic foci (Kruskal-Wallis test, $p < 0.01$).

(D–H) GSVA plots of highly variable GO terms (Kruskal-Wallis W test, $p < 0.01$) in models compared to fibroblastic foci.

(adjusted p value <0.05) differentially expressed genes (Figure 3A). When subjected to enrichment analysis, upregulated gene sets within the 3D spheroid model in the presence of TGF-β_1 included those associated with protein hydroxylation, peptidyl-lysyl hydroxylation, and ECM organization (Figures 3B–3E and Data S1). Genes upregulated within the 3D spheroid model when compared to the other model systems included fibrillar collagens (*COL3A1*) as well as genes previously proposed to have pathogenic roles in promoting progressive fibrosis through dysregulation of the ECM microenvironment including collagen prolyl hydroxylase activity (*P4HA3*), bone-type PYD collagen cross-linking (*PLOD2*), hyaluronan synthesis (*HAS1*), and pro-fibrotic fibroblast activation (*TWIST1*) (Figures 3E–3G).[22,39–41]

To further investigate the similarities and differences between each model system, we performed analysis of secreted cytokines within conditioned media from each model. We assayed cytokines and mediators previously proposed to have roles in lung fibrogenesis including modulators of chemotaxis, ECM remodeling, and inflammation.[42–57] From a recent large-scale proteomic analysis of IPF survival by Oldham et al.,[58] we identified those secreted factors significantly associated with an increased risk of death or transplant. Of the identified molecules, the 3D cell culture-based model had the greatest expression (TIMP-1 and VEGFA) or comparable expression (GDF-15, OPN, CCL2, POSTN, and TNC) of each secreted factor (Figure S1) compared with the other models.

The 3D spheroid model has similarities with the proteome of fibroblast foci

Having identified that the 3D spheroid model has the greatest similarity to the transcriptome of fibroblast foci and secreted proteins associated with increased risk of death, to further investigate the disease relevance of the model, we performed proteomic analyses of lung fibroblasts from patients with IPF cultured within the 3D spheroid model. We performed mass spectrometry (UPLC-HDMS^E) analysis of the 3D spheroid model and its secretome and compared this with a recently published dataset of the fibroblast focus proteome.[59] We identified diverse ECM proteins within the 3D spheroid model including multiple fibrillar collagens. Comparing the fibroblast focus proteome as quantified by Herrera et al.[59] to the 3D model, we identified a clear overlap with 88 ECM-associated proteins in common (Figure 4A and Data S2), with an interaction network for these proteins (Figure S2) identifying a complex ECM interactome and G:Profiler analysis (Figure 4B)

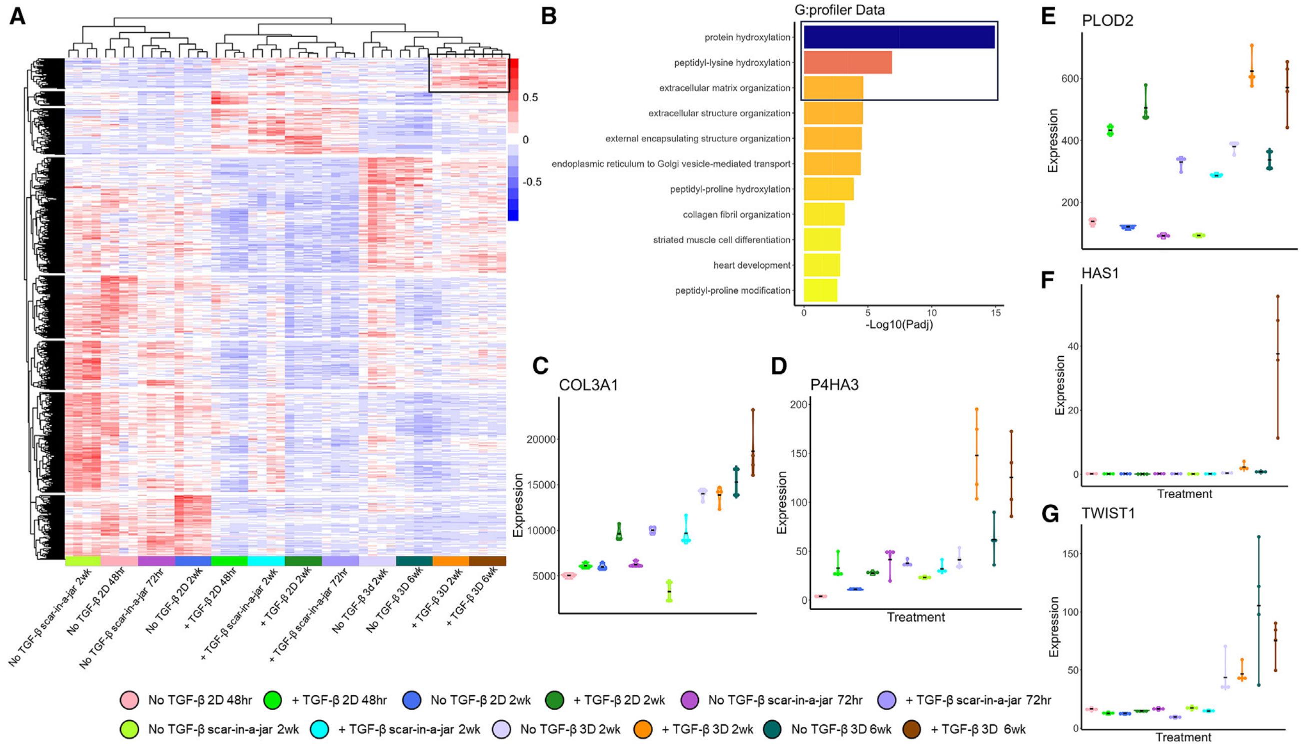

Figure 3. Clustering of RNA sequencing data from different fibroblast culture models reveals enrichment for ECM formation within the 3D spheroid model

(A) Heatmap showing hierarchical clustering of RNA sequencing (RNA-seq) data of the different fibroblast culture models. Genes shown are all differentially expressed between different models. Box shows upregulated genes within the 3D spheroid model used to identify GO terms in (B).

(B) G:profiler GO enrichment of genes associated with the 3D spheroid model (+TGF-β) (box in A). Box denoted GO terms discussed within the manuscript text.

(C–G) Violin plots of genes from (A) associated with regulation of the ECM microenvironment, (C) *COL3A1*, (D) *P4HA3*, (E) *PLOD2*, (F) *HAS1*, and (G) *TWIST1*.

identifying the most significant biological process Gene Ontology terms to include ECM organization and negative regulation of proteolysis. Additionally, we identified proteins that were only present in the 3D model or the fibroblast focus proteome (Figure 4C and Data S2). While this may partly reflect technical differences between the mass spectrometric methods used, proteins such as cartilage oligomeric matrix protein and periostin that were not reported within the fibroblast focus proteome were detected within the 3D model consistent with their mRNA expression within fibroblast foci in our spatial transcriptomics and with protein expression previously confirmed by others.[36,60,61]

Further comparison with the proteomic survival dataset of Oldham et al.[58] identified that 48 proteins present in the 3D spheroid model were significantly associated with an increased risk of death or transplant (Data S2). An interaction network for these risk proteins is shown in Figure 4D, with matrix metalloproteinases, elastin, and adhesion molecules serving as central hubs and pathway analysis (Figure 4E) identifying collagen-containing ECM to be the most enriched pathway.

Immunohistochemistry confirmed the expression of proteins identified within our spatial transcriptomic analyses (Figure 1) including tenascin-C (Figure 4F) as well as procollagen type 1 (Figure 4G) in comparable patterns between the 3D model and fibroblast foci (Figures 4F and 4G). Additionally, we confirmed the expression of proteins by a Luminex multiplex assay of cell-conditioned media including TIMP-1, periostin, and tenascin-C (Figure S1). Consistent with the 3D spheroid model recapitulating a complex ECM, transmission electron microscopy confirmed the incorporation of a complex 3D ECM including fibrillar collagens (Figures 4H and 4I).

The anti-LOXL2 antibody AB0023 does not inhibit LOXL2 catalytic activity, collagen cross-linking, or tissue stiffness in a disease-relevant model of fibrosis

Having confirmed that the 3D spheroid model recapitulates transcriptomic and protein characteristics of fibroblast foci, we proceeded to re-evaluate the potential of LOXL2 as a therapeutic target in lung fibrosis.

Previous studies have proposed that LOXL2 expression is increased within blood and lung tissue compartments of patients with lung fibrosis.[15,22,23,63] Consistent with these findings, we identified a significant increase in LOXL2 expression within serum sampled at time of diagnosis of patients with IPF when compared to control subjects (Figure 5A); while assessing *LOXL2* expression within our recently generated digital spatial transcriptome of human control and fibrotic lung tissue,[36] we identify significantly increased expression within fibroblast foci (Figure 5B). This finding is in keeping with our previous identification that the greatest expression of *LOXL2* is within

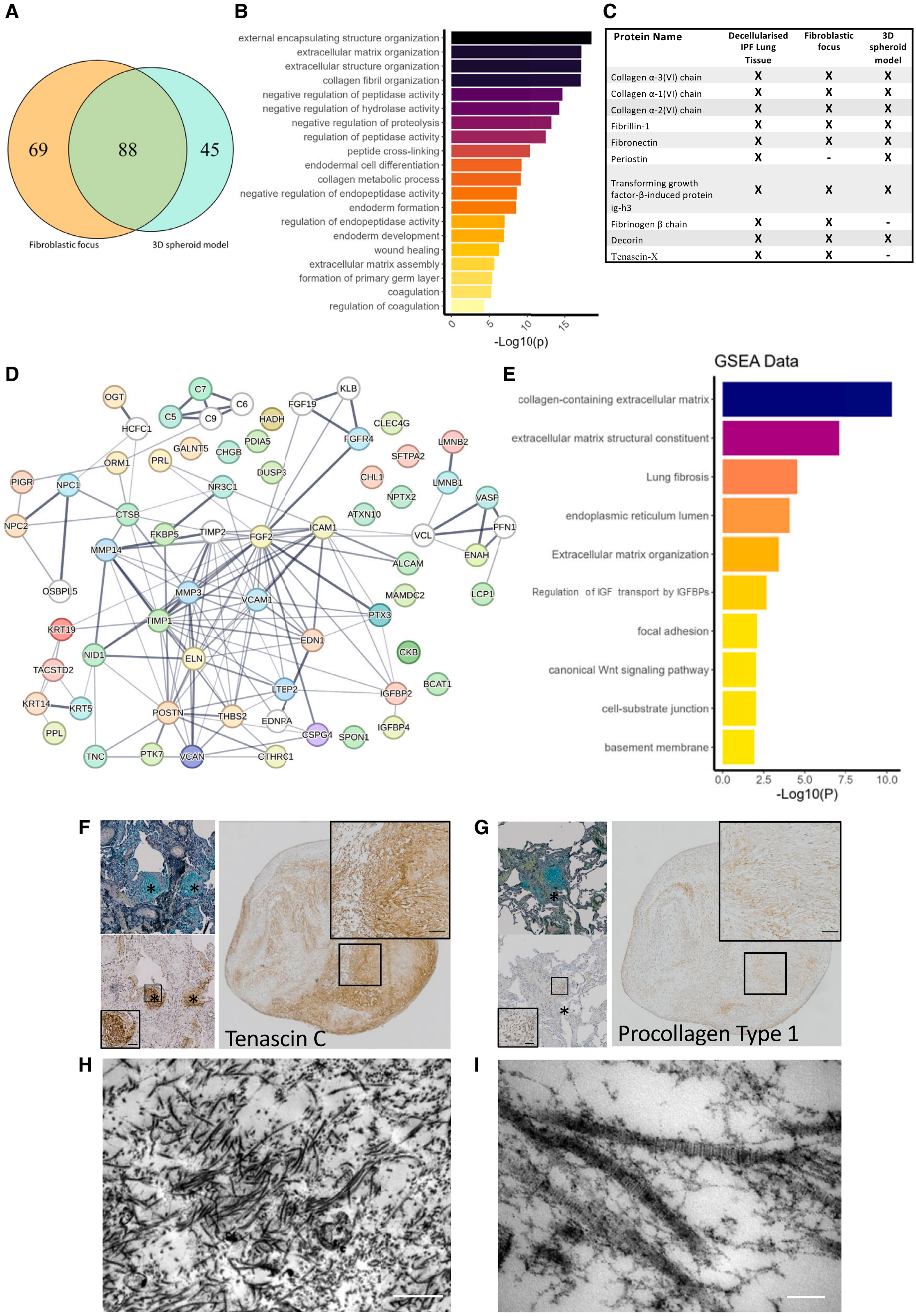

Protein Name	Decellularised IPF Lung Tissue	Fibroblastic focus	3D spheroid model
Collagen α-3(VI) chain	X	X	X
Collagen α-1(VI) chain	X	X	X
Collagen α-2(VI) chain	X	X	X
Fibrillin-1	X	X	X
Fibronectin	X	X	X
Periostin	X	-	X
Transforming growth factor-β-induced protein ig-h3	X	X	X
Fibrinogen β chain	X	X	-
Decorin	X	X	X
Tenascin-X	X	X	-

(legend on next page)

mesenchymal cells within fibroblast foci[22] and that *LOXL2* is co-expressed with *PLOD2*, which catalyzes telopeptide lysine hydroxylation to determine PYD collagen cross-linking. Using RNAscope *in situ* hybridization, we therefore confirmed *LOXL2* expression within the spheroid model and that this is co-expressed with *PLOD2* in a pattern comparable to that identified within fibroblast foci (Figure 5C).

We next compared the effects of the anti-LOXL2 antibody, AB0023 (a monoclonal antibody against human LOXL2 that was humanized to create simtuzumab),[26,27] within the 3D spheroid model using the small-molecule inhibitor PXS-S2A[64] as a positive control. We have previously identified that PXS-S2A at LOXL2-selective doses inhibits PYD collagen cross-linking and reduces tissue stiffness within the 3D spheroid model, confirming findings within an *in vivo* rat model of lung fibrosis driven by transient overexpression of active TGF-β_1 by adenoviral vector gene transfer.[15]

Lung fibroblasts from patients with IPF were cultured in the 3D spheroid model in the presence of AB0023 or an isotype control antibody at the same concentrations, as well as with PXS-S2A or its vehicle control. As previously reported,[15] after 6 weeks of culture, PXS-S2A significantly reduced PYD cross-linking (Figure 5D) and tissue stiffness (Figure 5E) without affecting total collagen content (Figure 5F). In contrast, there was no significant difference between AB0023 and the control antibody in PYD cross-linking, tissue stiffness, or collagen content (Figures 5D–5F). As we had found no effect of AB0023 upon collagen cross-linking or tissue stiffness, we measured LOXL2 catalytic activity within the conditioned media using an activity-based probe[65] identifying that AB0023 did not significantly inhibit LOXL2 catalytic activity while PXS-S2A did (Figure 5G). Nonetheless, in the presence of AB0023, we detected a decrease in VEGFA and fibronectin, two secreted proteins that have been reported as being regulated by LOXL2[66,67] (Figures 5H and 5I). In contrast, in the presence of PXS-S2A, we identified an increase in VEGFA and no effect on fibronectin, suggesting distinct mechanisms of action of PXS-S2A and AB0023, and that the observed effects of AB0023 are not via catalytic inhibition. Consistent with PXS-S2A having an antifibrotic effect, we identified a reduction in the expression of profibrotic genes including *COL3A1*, *PLOD2*, *P4HA3*, and *TWIST1* (Figure S3).

AB0023 is a potent inhibitor of angiogenesis

AB0023 is proposed to be an allosteric inhibitor of LOXL2 catalytic activity, with the binding epitope mapped to the scavenger receptor cysteine-rich (SRCR) domain 4 of human LOXL2.[27] Independent of catalytic amine oxidase activity, the SRCR domains of LOXL2 have been reported to promote angiogenesis.[68] As AB0023 has previously been reported in preclinical cancer studies to be anti-angiogenic,[69] we investigated the functional effect of the antibody on angiogenesis to exclude the possibility that our inability to detect any significant effect of AB0023 on LOXL2 catalytic activity, PYD collagen cross-linking, and tissue stiffness was not due to the loss of antibody efficacy.

We therefore evaluated the effect of AB0023 within a human endothelial tube formation assay.[70] In the presence of AB0023, we observed a significant decrease in tube formation (Figure 6A), with quantification of topological parameters identifying a significant decrease in number of loops, number of branching points, and total tube length (Figures 6B–6D). In contrast, the small-molecule inhibitor PXS-S2A did not significantly decrease measures of tube formation, consistent with distinct mechanisms of action of these two agents (Figure S4).

Together, these results identify that while AB0023 is a potent inhibitor of angiogenesis, it does not significantly inhibit LOXL2 catalytic activity, collagen cross-linking, or tissue stiffness in a human disease-relevant model of fibrosis.

DISCUSSION

Tissue from patients with lung fibrosis shows marked heterogeneity of pathological changes which challenges its study and modeling. Fibroblast foci are considered the site of active fibrogenesis; however, until recently, their microenvironment has been poorly understood. Here, we identify that an *in vitro* 3D spheroid model closely resembles the transcriptome of human fibroblast foci, further confirming disease relevance through proteomic comparisons. We then investigated within the model whether the anti-LOXL2 monoclonal antibody AB0023 could inhibit the ability of LOXL2 to catalyze collagen cross-linking and reduce tissue stiffness. In these assays, AB0023 did not inhibit catalytic activity, collagen cross-linking, or reduce tissue stiffness; however, in endothelial tube formation assays, it was a potent inhibitor of angiogenesis, consistent with a mechanism

Figure 4. The 3D spheroid model recapitulates features of the complex ECM structure identified within fibroblast foci
(A) Venn diagram showing the number of unique and common ECM proteins identified between the fibroblast focus spatial proteome (Herrera et al.[59]) and the 3D spheroid model.
(B) G:Profiler analysis of 88 ECM proteins common between the fibroblastic focus spatial proteome and the 3D spheroid model proteome. Top 20 most significant biological process GO terms shown.
(C) A comparison of proteomic data derived from the ECM of IPF tissue (Booth et al.[62]), the fibroblast focus spatial proteome (Herrera et al.), and the 3D spheroid model, showing the top ECM proteins by abundance in IPF ECM and their presence or absence in the other datasets. X indicates proteins that are present and those that are absent.
(D) STRING map showing proteins (Data S2C) identified within the 3D spheroid model that have a hazard ratio >1 in Oldham et al.[58] for increased risk for death or lung transplantation. Each protein has up to 10 medium confidence interaction partners.
(E) G:Profiler analysis showing top 10 pathways from proteins identified in (D).
(F and G) Immunohistochemical staining of IPF lung tissue and 3D spheroids. Top left is a Masson's trichrome stain of IPF tissue with a fibroblast focus identified by *, with a serial section bottom left with immunohistochemical staining for tenascin C (F) or procollagen type 1 (G); on the right-hand side is the corresponding immunohistochemical staining for the 3D spheroid model with a higher magnification inset. Inset scale bars are 50 μm.
(H and I) Transmission electron microscopy images of the 3D spheroid model identifying (H) a complex intercalated ECM and (I) the D-banding characteristic of fibrillar collagen. Scale bars are 1 μm (H) and 100 nm (I).

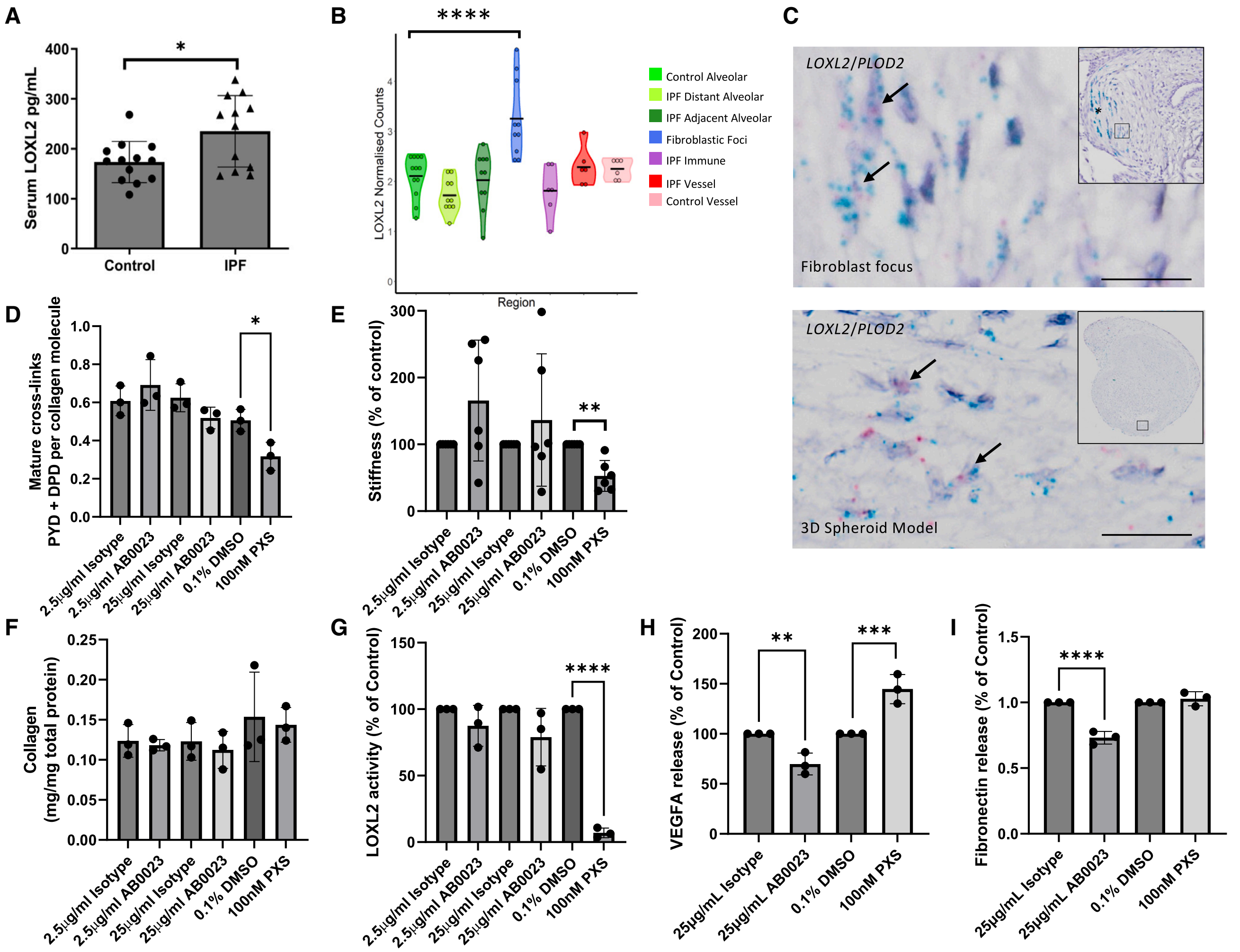

Figure 5. The anti-LOXL2 antibody AB0023 does not inhibit collagen-cross linking, tissue stiffness, or catalytic amine oxidase activity in a disease-relevant model of fibrosis

(A) LOXL2 protein levels were assayed in the serum of patients with IPF (n = 12) and control subjects (n = 13).

(B) Expression of *LOXL2* within spatially resolved regions of control and IPF lung tissue. Data from Eyres et al.[30]

(C) Representative images of mRNA expression of *LOXL2* (green chromagen) and *PLOD2* (red chromagen) within a fibroblast focus (*) of IPF lung tissue and the 3D spheroid model using RNAscope RNA *in situ* hybridization. Scale bars are 20 μm. Arrows indicating cells with co-expression of *LOXL2* and *PLOD2*.

(D–I) Lung fibroblasts from patients with IPF (n = 3 donors across 2 independent experiments) were used in the 3D spheroid model in the presence of AB0023 or an isotype control antibody at the same concentrations, as well as with PXS-S2A or its vehicle control (0.1% DMSO).

(D) Total mature trivalent (PYD + DPD) collagen cross-links determined by ELISA (n = 3).

(E) Tissue stiffness measured from parallel-plate compression testing determined by Young's modulus and represented as a proportion of control (n = 6).

(F) Total collagen content determined by hydroxyproline assay.

(G) LOXL2 catalytic amine oxidase activity within the conditioned media was assessed using an activity-based probe (n = 3).

(H) VEGFA within cell-conditioned media determined by ELISA.

(I) Fibronectin within cell-conditioned media determined by ELISA.

Data are mean ± SD. *p < 0.05, **p < 0.01, ***p < 0.001, ****p < 0.0001. Unpaired two-tailed t test (A) (t = 2.651, degrees of freedom = 23), Wilcoxon test with Benjamini-Hochberg multiple test correction (B), and ANOVA with Šídák's multiple comparisons test (D–H) were used to evaluate statistical significance (F values: (D) 7.508, (E) 2.862, (F) 0.8966, (G) 33.06, (H) 34.1, and (I) 45.03. Degrees of freedom: (D) 12, (E) 30, (F) 12, (G) 12, (H) 12, and (I) 12). Error bars are standard deviation.

of action independent of lysyl amine oxidase inhibition. These findings have relevance to the interpretation of the failure of simtuzumab/AB0023 to demonstrate efficacy in clinical trials of fibrotic diseases where it was expected to inhibit collagen cross-linking and reduce tissue stiffening.

The importance of human disease-relevant models of complex diseases such as lung fibrogenesis is increasingly proposed for preclinical studies where drug attrition rates are high and translation is challenging.[32] However, while different cell model systems have been utilized, there has been limited validation.

CellPress
OPEN ACCESS

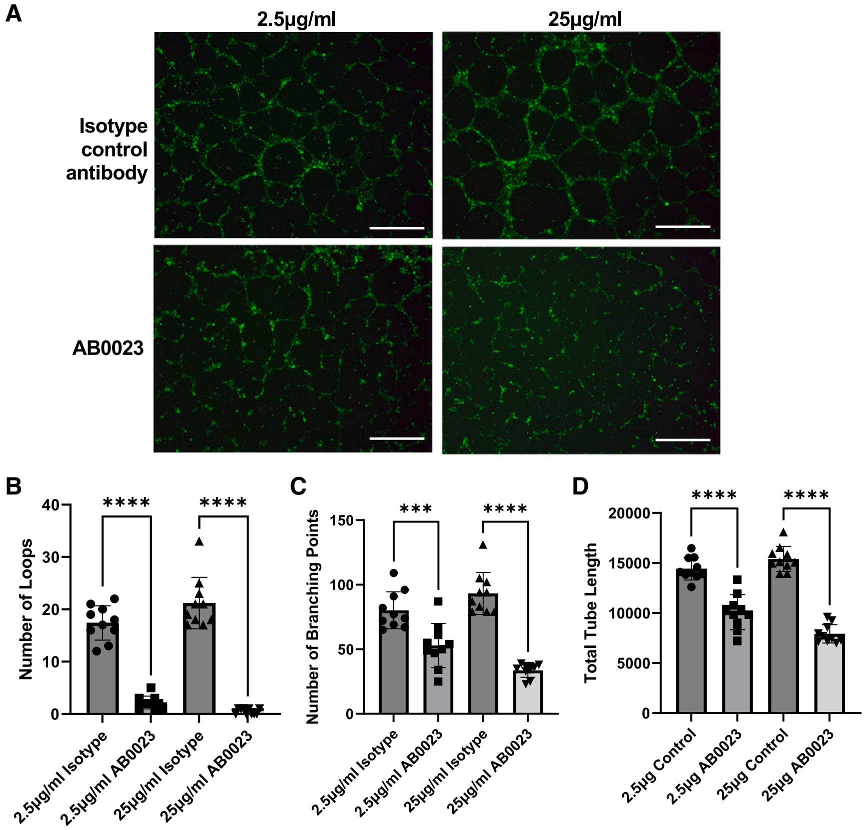

Figure 6. The anti-LOXL2 antibody AB0023 is a potent inhibitor of angiogenesis

Endothelial tube formation by human umbilical vein endothelial cells was assessed in the presence of AB0023 or an isotype control antibody at the same concentration (n = 10 replicates per condition across 3 independent experiments).

(A) Representative images of endothelial tube formation under each condition visualized by calcein staining. Scale bars are 500 μm.

(B–D) Quantification of topological parameters of capillary structure by computer-aided image analysis for (B) number of loops, (C) number of branching points, and (D) total tube length. Data are mean ± SD. *$p < 0.05$, **$p < 0.01$, ***$p < 0.001$, ****$p < 0.0001$. ANOVA with Šídák's multiple comparisons test was used to determine statistical significance (F statistics: B 120.3, C 36.31, and D 74.56. Degrees of freedom: 36). Error bars are standard deviation.

Within human lung fibrogenesis, the fibroblast focus represents a key site of disease activity. Here, advances in spatial biology approaches provided the opportunity to better characterize fibroblast foci and compare the relevant human disease microenvironment with model systems. Bioinformatic analysis of standard 2D cell culture, the Scar-in-a-Jar model, and the 3D spheroid model identified that the 3D spheroid model has significant transcriptomic similarity with fibroblast foci. These data support the importance of 3D models incorporating ECM to understand biological phenomena.[71–73] The finding of comparable expression patterns within the model to human lung fibrogenesis suggests that mechanistic and therapeutic studies within the model have translational relevance to human lung fibrosis. Consistent with this, we have recently demonstrated within the 3D spheroid model that the promotion of PYD collagen cross-linking recapitulates key features identified within human lung fibrosis tissue including altered collagen nanoarchitecture and increased tissue stiffness.[15,22] Our identification of enrichment of a skeletal development signature within fibroblastic foci is consistent with the shift from a skin (allysine-derived) to a bone type (hydroxyallysine-derived) of collagen cross-link being of pathogenetic importance in human lung fibrosis. While our studies have focused on the recapitulation of fibroblast foci, the ongoing rapid advances in high-resolution spatial omics approaches provide the possibility of future refinement of this approach to include other disease-relevant cell types within the entire fibrotic niche.

Human tissue and blood analyses as well as preclinical *in vitro* and *in vivo* studies have implicated LOXL2 as a core mediator of human fibrogenesis, with LOXL2-mediated collagen cross-linking increasing tissue stiffness and promoting fibrosis progression. However, the failure of simtuzumab/AB0023 to demonstrate efficacy in clinical trials of fibrotic diseases has raised uncertainty regarding the relevance of LOXL2 as a therapeutic target. AB0023 was generated by immunization of mice using human LOXL2 protein and characterized as a partial non-competitive allosteric inhibitor of LOXL2 catalytic activity.[27] A subsequent *in vivo* preclinical study of fibrosis and cancer reported that there was a significant reduction in collagen cross-linking following AB0023 treatment,[20] consistent with AB0023 acting via inhibition of catalytic activity. However, no direct biochemical quantification of collagen cross-linking was performed, with the reduction in collagen cross-linking inferred through quantification in the signal of polarized Sirius Red staining of tissue samples. A strong linear anionic dye, Sirius Red, stains collagen by reacting, via its sulfonic acid groups, with basic groups present in the collagen molecule, enhancing their natural birefringence under cross-polarized light.[74,75] While Sirius Red staining under cross-polarized light has been widely adopted as a method to assess collagen tissue deposition patterns, to our knowledge, no relationship between Sirius Red quantification and collagen cross-linking density has been demonstrated. In keeping with this in our own second harmonic generation (SHG) study of collagen macro/supramolecular changes following collagen cross-linking modulation, we identified that no SHG signature was directly associated with cross-linking density.[76] Thus, in the absence of direct measurement of target engagement (i.e., inhibition of collagen cross-linking activity), the efficacy of AB0023 in inhibiting collagen cross-linking and pathologic tissue stiffness remained uncertain.

We identified that AB0023 did not significantly inhibit LOXL2 catalytic activity, collagen cross-linking, or tissue stiffness within a human disease-relevant model of fibrosis. In support of this, a recent study identified that AB0023 is only a low potency, partial inhibitor of LOXL2 activity within cell-free assays, while LOXL2 enzymatic activity in human plasma cannot be inhibited by AB0023.[65] The C-terminal domain of LOXL2 contains the active enzyme region, which is highly conserved between LOX family members, while four SRCR domains make up the amino-terminal domain of LOXL2, with the binding epitope of AB0023 mapped to SRCR domain 4.[27] SRCR domains are found on secreted and cell surface-bound proteins and are proposed to be involved in cell adhesion and signaling.[77] A number of studies have identified evidence for SRCR-mediated roles of LOXL2 independent of catalytic domain activity including angiogenesis and keratinocyte differentiation.[67,68,78]

Consistent with previous preclinical cancer reports that AB0023 is anti-angiogenic and anti-metastatic, we identified a significant reduction in VEGFA and fibronectin within the conditioned media of 3D spheroids treated with AB0023. It has previously been reported that, independent of the catalytic domain, the SRCR domains of LOXL2, to which AB0023 binds, regulate VEGFA transcription and promote VEGFA secretion and angiogenesis.[67] Furthermore, it has previously been shown to inhibit VEGF-induced phosphorylation of ERK1/2 in endothelial cells.[69] Thus, while AB0023 does not significantly inhibit LOXL2 catalytic activity or collagen cross-linking, it is a potent inhibitor of angiogenesis which may account for its effects in animal models. Together, these findings suggest the effect of AB0023 is SRCR domain mediated and independent of LOXL2 catalytic inhibition.

In conclusion, we identified that an *in vitro* 3D spheroid model most closely resembles the transcriptome of human fibroblast foci, further confirming disease relevance through proteomic analyses. Within this model, the anti-LOXL2 antibody AB0023 did not inhibit the catalytic activity of LOXL2 or matrix stiffness; in contrast, selective targeting of LOXL2 catalytic activity using a small-molecule inhibitor significantly reduced these endpoints. These findings inform the failure of simtuzumab to demonstrate anti-fibrotic efficacy in clinical trials of fibrotic diseases where the expectation was to target collagen cross-linking and reduce tissue stiffening.

Limitations of the study

This study has a number of limitations. Firstly, while we have focused upon a key feature of human lung fibrogenesis—fibroblast foci—the lung fibrosis microenvironment is complex and includes multiple cell populations. Future investigations will consider the larger microenvironment and the potential to further leverage spatial omics studies to incorporate additional cell types within the 3D spheroid model, further recapitulating the human lung fibrosis microenvironment *in vitro*. Secondly, we have not provided direct evidence that AB0023 is acting directly via the SRCR domains of LOXL2, with future studies required to further dissect the non-catalytic roles of LOXL2.

STAR★METHODS

Detailed methods are provided in the online version of this paper and include the following:

- KEY RESOURCES TABLE
- RESOURCE AVAILABILITY
  - Lead contact
  - Materials availability
  - Data and code availability
- EXPERIMENTAL MODEL AND SUBJECT DETAILS
  - Clinical samples
  - Parenchymal lung fibroblast cultures
  - Primary human umbilical vein endothelial cells (HUVEC)
- METHOD DETAILS
  - Spatial transcriptomic data analyses
  - Carmine red staining of fixed spheroids
  - RNAseq and bioinformatic analyses
  - Proteomic analysis
  - Luminex analysis
  - Immunohistochemistry
  - Transmission electron microscopy (TEM)
  - LOXL2 serum assay
  - RNA in situ hybridisation
  - Evaluation of AB0023 in the 3D spheroid model
  - LOXL2 activity assay
  - Protein, hydroxyproline and collagen cross-link analysis
  - Parallel plate compression testing
  - Enzyme-linked immuno-sorbent assay
  - *In vitro* human umbilical vein endothelial cell assay
  - Quantitative real-time PCR (qPCR)
- QUANTIFICATION AND STATISTICAL ANALYSIS

SUPPLEMENTAL INFORMATION

Supplemental information can be found online at https://doi.org/10.1016/j.xcrm.2024.101695.

ACKNOWLEDGMENTS

This project was supported by NC3Rs (NC/V002384/1), the Medical Research Council (MR/S025480/1 and MR/Y002989/1), the Wellcome Trust (100638/Z/12/Z), and the AAIR Charity. C.J.B. and L.W. acknowledge the support of the NIHR Southampton Biomedical Research Centre. F.C. was supported by Medical Research Foundation (MRF-091–0003-RG-CONFO). The graphical abstract and the project design schematic in Figure 2A were created with BioRender.com. We thank Carine Fixmer, Maria Lane, Benjamin Johnson, and the nurses of the Southampton Biomedical Research Unit for their help in the collection of human samples, supported by the Wessex Clinical Research Network and the National Institute for Health Research, UK. We thank Wolfgang Jarolimek at Pharmaxis Ltd. for provision of PXS-S2A and supporting LOXL2 activity assays. We thank Gilead Sciences, Inc. for provision of the AB0023 and isotype control reagents.

AUTHOR CONTRIBUTIONS

J.A.B., D.E.D., and M.G.J. conceived and designed the study. J.A.B., E.R.D., C.J.B., L.W., J.J.W.R., K.L., E.O., R.A.R., F.C., L.N.S., and J.C. performed all experiments and data analysis. D.A.J. supported confocal imaging studies. P.D.M. and Y.W. supported data analysis. A.V.P. supported bioinformatic analysis. S.J., B.G.M., and A.A. supported tissue collection. P.S. supported mass spectrometry analysis. M.V. and N.K. supported laser-capture data studies. A.F. supported pathology review and interpretation. All authors read and approved the manuscript.

DECLARATION OF INTERESTS

M.G.J., D.E.D., and Y.W. acknowledge grants from Boehringer Ingelheim. D.E.D. is co-founder of, shareholder in, and consultant to Synairgen Research Ltd. M.G.J. acknowledges consultancy fees from Skyhawk Therapeutics. J.J.W.R., K.L., and P.D.M. are current employees of Synairgen Research

CellPress
OPEN ACCESS

Ltd. L.R. acknowledges grants from Boehringer Ingelheim and consultancy fees from Biogen, Celgene, Boehringer Ingelheim, Roche, Pliant Therapeutics, BMS, CSL Behring, FibroGen, Veracyte, and Chiesi.

Received: July 10, 2023
Revised: February 26, 2024
Accepted: July 29, 2024
Published: August 21, 2024

REFERENCES

1. Richeldi, L., Collard, H.R., and Jones, M.G. (2017). Idiopathic pulmonary fibrosis. Lancet *389*, 1941–1952. https://doi.org/10.1016/S0140-6736(17)30866-8.
2. Wipff, P.-J., Rifkin, D.B., Meister, J.-J., and Hinz, B. (2007). Myofibroblast contraction activates latent TGF-beta1 from the extracellular matrix. J. Cell Biol. *179*, 1311–1323. https://doi.org/10.1083/jcb.200704042.
3. Liu, F., Mih, J.D., Shea, B.S., Kho, A.T., Sharif, A.S., Tager, A.M., and Tschumperlin, D.J. (2010). Feedback amplification of fibrosis through matrix stiffening and COX-2 suppression. J. Cell Biol. *190*, 693–706. https://doi.org/10.1083/jcb.201004082.
4. Zhou, Y., Huang, X., Hecker, L., Kurundkar, D., Kurundkar, A., Liu, H., Jin, T.-H., Desai, L., Bernard, K., and Thannickal, V.J. (2013). Inhibition of mechanosensitive signaling in myofibroblasts ameliorates experimental pulmonary fibrosis. J. Clin. Invest. *123*, 1096–1108. https://doi.org/10.1172/JCI66700.
5. Parker, M.W., Rossi, D., Peterson, M., Smith, K., Sikström, K., White, E.S., Connett, J.E., Henke, C.A., Larsson, O., and Bitterman, P.B. (2014). Fibrotic extracellular matrix activates a profibrotic positive feedback loop. J. Clin. Invest. *124*, 1622–1635. https://doi.org/10.1172/JCI71386.
6. Tschumperlin, D.J., Ligresti, G., Hilscher, M.B., and Shah, V.H. (2018). Mechanosensing and fibrosis. J. Clin. Invest. *128*, 74–84. https://doi.org/10.1172/JCI93561.
7. Herrera, J., Henke, C.A., and Bitterman, P.B. (2018). Extracellular matrix as a driver of progressive fibrosis. J. Clin. Invest. *128*, 45–53. https://doi.org/10.1172/JCI93557.
8. Jones, M.G., Fabre, A., Schneider, P., Cinetto, F., Sgalla, G., Mavrogordato, M., Jogai, S., Alzetani, A., Marshall, B.G., O'Reilly, K.M.A., et al. (2016). Three-dimensional characterization of fibroblast foci in idiopathic pulmonary fibrosis. JCI Insight *1*, e86375. https://doi.org/10.1172/jci.insight.86375.
9. Fiore, V.F., Wong, S.S., Tran, C., Tan, C., Xu, W., Sulchek, T., White, E.S., Hagood, J.S., and Barker, T.H. (2018). αvβ3 Integrin drives fibroblast contraction and strain stiffening of soft provisional matrix during progressive fibrosis. JCI Insight *3*, e97597. https://doi.org/10.1172/jci.insight.97597.
10. Herrera, J., Forster, C., Pengo, T., Montero, A., Swift, J., Schwartz, M.A., Henke, C.A., and Bitterman, P.B. (2019). Registration of the extracellular matrix components constituting the fibroblastic focus in idiopathic pulmonary fibrosis. JCI Insight *4*, e125185. https://doi.org/10.1172/jci.insight.125185.
11. King, T.E., Schwarz, M.I., Brown, K., Tooze, J.A., Colby, T.V., Waldron, J.A., Flint, A., Thurlbeck, W., and Cherniack, R.M. (2001). Idiopathic Pulmonary Fibrosis. Am. J. Respir. Crit. Care Med. *164*, 1025–1032. https://doi.org/10.1164/ajrccm.164.6.2001056.
12. Flaherty, K.R., Colby, T.V., Travis, W.D., Toews, G.B., Mumford, J., Murray, S., Thannickal, V.J., Kazerooni, E.A., Gross, B.H., Lynch, J.P., and Martinez, F.J. (2003). Fibroblastic Foci in Usual Interstitial Pneumonia. Am. J. Respir. Crit. Care Med. *167*, 1410–1415. https://doi.org/10.1164/rccm.200204-373OC.
13. Harada, T., Watanabe, K., Nabeshima, K., Hamasaki, M., and Iwasaki, H. (2013). Prognostic significance of fibroblastic foci in usual interstitial pneumonia and non-specific interstitial pneumonia. Respirology *18*, 278–283. https://doi.org/10.1111/j.1440-1843.2012.02272.x.
14. Senior, R.M., Bielefeld, D.R., and Abensohn, M.K. (1975). The Effects of Proteolytic Enzymes on the Tensile Strength of Human Lung. Am. Rev. Respir. Dis. *111*, 184–188. https://doi.org/10.1164/arrd.1975.111.2.184.
15. Jones, M.G., Andriotis, O.G., Roberts, J.J., Lunn, K., Tear, V.J., Cao, L., Ask, K., Smart, D.E., Bonfanti, A., Johnson, P., et al. (2018). Nanoscale dysregulation of collagen structure-function disrupts mechano-homeostasis and mediates pulmonary fibrosis. Elife *7*, e36354. https://doi.org/10.7554/eLife.36354.
16. Shoulders, M.D., and Raines, R.T. (2009). COLLAGEN STRUCTURE AND STABILITY. Annu. Rev. Biochem. *78*, 929–958. https://doi.org/10.1146/annurev.biochem.77.032207.120833.
17. Kadler, K.E., Holmes, D.F., Trotter, J.A., and Chapman, J.A. (1996). Collagen fibril formation. Biochem. J. *316*, 1–11.
18. Yamauchi, M., and Sricholpech, M. (2012). Lysine post-translational modifications of collagen. Essays Biochem. *52*, 113–133. https://doi.org/10.1042/bse0520113.
19. Trackman, P.C. (2016). Lysyl Oxidase Isoforms and Potential Therapeutic Opportunities for Fibrosis and Cancer. Expert Opin. Ther. Targets *20*, 935–945. https://doi.org/10.1517/14728222.2016.1151003.
20. Barry-Hamilton, V., Spangler, R., Marshall, D., McCauley, S., Rodriguez, H.M., Oyasu, M., Mikels, A., Vaysberg, M., Ghermazien, H., Wai, C., et al. (2010). Allosteric inhibition of lysyl oxidase–like-2 impedes the development of a pathologic microenvironment. Nat. Med. *16*, 1009–1017. https://doi.org/10.1038/nm.2208.
21. Yang, J., Savvatis, K., Kang, J.S., Fan, P., Zhong, H., Schwartz, K., Barry, V., Mikels-Vigdal, A., Karpinski, S., Kornyeyev, D., et al. (2016). Targeting LOXL2 for cardiac interstitial fibrosis and heart failure treatment. Nat. Commun. *7*, 13710. https://doi.org/10.1038/ncomms13710.
22. Brereton, C.J., Yao, L., Davies, E.R., Zhou, Y., Vukmirovic, M., Bell, J.A., Wang, S., Ridley, R.A., Dean, L.S.N., Andriotis, O.G., et al. (2022). Pseudohypoxic HIF pathway activation dysregulates collagen structure-function in human lung fibrosis. Elife *11*, e69348. https://doi.org/10.7554/eLife.69348.
23. Chien, J.W., Richards, T.J., Gibson, K.F., Zhang, Y., Lindell, K.O., Shao, L., Lyman, S.K., Adamkewicz, J.I., Smith, V., Kaminski, N., and O'Riordan, T. (2014). Serum lysyl oxidase-like 2 levels and idiopathic pulmonary fibrosis disease progression. Eur. Respir. J. *43*, 1430–1438. https://doi.org/10.1183/09031936.00141013.
24. Harrison, S.A., Abdelmalek, M.F., Caldwell, S., Shiffman, M.L., Diehl, A.M., Ghalib, R., Lawitz, E.J., Rockey, D.C., Schall, R.A., Jia, C., et al. (2018). Simtuzumab Is Ineffective for Patients With Bridging Fibrosis or Compensated Cirrhosis Caused by Nonalcoholic Steatohepatitis. Gastroenterology *155*, 1140–1153. https://doi.org/10.1053/j.gastro.2018.07.006.
25. Muir, A.J., Levy, C., Janssen, H.L.A., Montano-Loza, A.J., Shiffman, M.L., Caldwell, S., Luketic, V., Ding, D., Jia, C., McColgan, B.J., et al. (2019). Simtuzumab for Primary Sclerosing Cholangitis: Phase 2 Study Results With Insights on the Natural History of the Disease. Hepatology *69*, 684–698. https://doi.org/10.1002/hep.30237.
26. Raghu, G., Brown, K.K., Collard, H.R., Cottin, V., Gibson, K.F., Kaner, R.J., Lederer, D.J., Martinez, F.J., Noble, P.W., Song, J.W., et al. (2017). Efficacy of simtuzumab versus placebo in patients with idiopathic pulmonary fibrosis: a randomised, double-blind, controlled, phase 2 trial. Lancet Respir. Med. *5*, 22–32. https://doi.org/10.1016/S2213-2600(16)30421-0.
27. Rodriguez, H.M., Vaysberg, M., Mikels, A., McCauley, S., Velayo, A.C., Garcia, C., and Smith, V. (2010). Modulation of Lysyl Oxidase-like 2 Enzymatic Activity by an Allosteric Antibody Inhibitor. J. Biol. Chem. *285*, 20964–20974. https://doi.org/10.1074/jbc.M109.094136.
28. Jenkins, R.G., Moore, B.B., Chambers, R.C., Eickelberg, O., Königshoff, M., Kolb, M., Laurent, G.J., Nanthakumar, C.B., Olman, M.A., Pardo, A., et al. (2017). An Official American Thoracic Society Workshop Report: Use of Animal Models for the Preclinical Assessment of Potential Therapies for Pulmonary Fibrosis. Am. J. Respir. Cell Mol. Biol. *56*, 667–679. https://doi.org/10.1165/rcmb.2017-0096ST.

29. Tashiro, J., Rubio, G.A., Limper, A.H., Williams, K., Elliot, S.J., Ninou, I., Aidinis, V., Tzouvelekis, A., and Glassberg, M.K. (2017). Exploring Animal Models That Resemble Idiopathic Pulmonary Fibrosis. Front. Med. *4*, 118. https://doi.org/10.3389/fmed.2017.00118.

30. Jones, M.G., Fletcher, S., and Richeldi, L. (2013). Idiopathic Pulmonary Fibrosis: Recent Trials and Current Drug Therapy. RES *86*, 353–363. https://doi.org/10.1159/000356958.

31. Decaris, M.L., Gatmaitan, M., FlorCruz, S., Luo, F., Li, K., Holmes, W.E., Hellerstein, M.K., Turner, S.M., and Emson, C.L. (2014). Proteomic Analysis of Altered Extracellular Matrix Turnover in Bleomycin-induced Pulmonary Fibrosis. Mol. Cell. Proteomics *13*, 1741–1752. https://doi.org/10.1074/mcp.M113.037267.

32. Loewa, A., Feng, J.J., and Hedtrich, S. (2023). Human disease models in drug development. Nat. Rev. Bioeng. *1*, 1–15. https://doi.org/10.1038/s44222-023-00063-3.

33. Madhusoodanan, J. (2019). Matrix mimics shape cell studies. Nature *566*, 563–565. https://doi.org/10.1038/d41586-019-00681-1.

34. Sacchi, M., Bansal, R., and Rouwkema, J. (2020). Bioengineered 3D Models to Recapitulate Tissue Fibrosis. Trends Biotechnol. *38*, 623–636. https://doi.org/10.1016/j.tibtech.2019.12.010.

35. Marx, V. (2021). Method of the Year: spatially resolved transcriptomics. Nat. Methods *18*, 9–14. https://doi.org/10.1038/s41592-020-01033-y.

36. Eyres, M., Bell, J.A., Davies, E.R., Fabre, A., Alzetani, A., Jogai, S., Marshall, B.G., Johnston, D.A., Xu, Z., Fletcher, S.V., et al. (2022). Spatially resolved deconvolution of the fibrotic niche in lung fibrosis. Cell Rep. *40*, 111230. https://doi.org/10.1016/j.celrep.2022.111230.

37. Conforti, F., Ridley, R., Brereton, C., Alzetani, A., Johnson, B., Marshall, B.G., Fletcher, S.V., Ottensmeier, C.H., Richeldi, L., Skipp, P., et al. (2020). Paracrine SPARC signaling dysregulates alveolar epithelial barrier integrity and function in lung fibrosis. Cell Death Dis. *6*, 54. https://doi.org/10.1038/s41420-020-0289-9.

38. Chen, C.Z.C., Peng, Y.X., Wang, Z.B., Fish, P.V., Kaar, J.L., Koepsel, R.R., Russell, A.J., Lareu, R.R., and Raghunath, M. (2009). The Scar-in-a-Jar: studying potential antifibrotic compounds from the epigenetic to extracellular level in a single well. Br. J. Pharmacol. *158*, 1196–1209. https://doi.org/10.1111/j.1476-5381.2009.00387.x.

39. Yeo, S.-Y., Lee, K.-W., Shin, D., An, S., Cho, K.-H., and Kim, S.-H. (2018). A positive feedback loop bi-stably activates fibroblasts. Nat. Commun. *9*, 3016. https://doi.org/10.1038/s41467-018-05274-6.

40. Luo, Y., Xu, W., Chen, H., Warburton, D., Dong, R., Qian, B., Selman, M., Gauldie, J., Kolb, M., and Shi, W. (2015). A novel profibrotic mechanism mediated by TGF-β-stimulated collagen prolyl hydroxylase expression in fibrotic lung mesenchymal cells. J. Pathol. *236*, 384–394. https://doi.org/10.1002/path.4530.

41. Valenzi, E., Bahudhanapati, H., Tan, J., Tabib, T., Sullivan, D.I., Nouraie, M., Sembrat, J., Fan, L., Chen, K., Liu, S., et al. (2023). Single-Nucleus Chromatin Accessibility Identifies a Critical Role for TWIST1 in IPF Myofibroblast Activity. Eur. Respir. J. *62*, 2200474. https://doi.org/10.1183/13993003.00474-2022.

42. Radwanska, A., Cottage, C.T., Piras, A., Overed-Sayer, C., Sihlbom, C., Budida, R., Wrench, C., Connor, J., Monkley, S., Hazon, P., et al. (2022). Increased expression and accumulation of GDF15 in IPF extracellular matrix contribute to fibrosis. JCI Insight *7*, e153058. https://doi.org/10.1172/jci.insight.153058.

43. Almuntashiri, S., Alhumaid, A., Zhu, Y., Han, Y., Dutta, S., Khilji, O., Zhang, D., and Wang, X. (2023). TIMP-1 and its potential diagnostic and prognostic value in pulmonary diseases. Chin. Med. J. Pulm. Crit. Care Med. *1*, 67–76. https://doi.org/10.1016/j.pccm.2023.05.002.

44. Estany, S., Vicens-Zygmunt, V., Llatjós, R., Montes, A., Penín, R., Escobar, I., Xaubet, A., Santos, S., Manresa, F., Dorca, J., and Molina-Molina, M. (2014). Lung fibrotic tenascin-C upregulation is associated with other extracellular matrix proteins and induced by TGFβ1. BMC Pulm. Med. *14*, 120. https://doi.org/10.1186/1471-2466-14-120.

45. Yoshihara, T., Nanri, Y., Nunomura, S., Yamaguchi, Y., Feghali-Bostwick, C., Ajito, K., Murakami, S., Mawatari, M., and Izuhara, K. (2020). Periostin plays a critical role in the cell cycle in lung fibroblasts. Respir. Res. *21*, 38. https://doi.org/10.1186/s12931-020-1299-0.

46. Zhu, X., Ji, J., and Han, X. (2024). Osteopontin: an essential regulatory protein in idiopathic pulmonary fibrosis. J. Mol. Histol. *55*, 1–13. https://doi.org/10.1007/s10735-023-10169-y.

47. Atamas, S.P. (2017). Vascular Endothelial Growth Factor in Idiopathic Pulmonary Fibrosis. An Imbalancing Act. Am. J. Respir. Crit. Care Med. *196*, 409–411. https://doi.org/10.1164/rccm.201703-0605ED.

48. Suga, M., Iyonaga, K., Ichiyasu, H., Saita, N., Yamasaki, H., and Ando, M. (1999). Clinical significance of MCP-1 levels in BALF and serum in patients with interstitial lung diseases. Eur. Respir. J. *14*, 376–382. https://doi.org/10.1034/j.1399-3003.1999.14b23.x.

49. Liu, S., Liu, C., Wang, Q., Liu, S., and Min, J. (2023). CC Chemokines in Idiopathic Pulmonary Fibrosis: Pathogenic Role and Therapeutic Potential. Biomolecules *13*, 333. https://doi.org/10.3390/biom13020333.

50. Russo, R.C., Alessandri, A.L., Garcia, C.C., Cordeiro, B.F., Pinho, V., Cassali, G.D., Proudfoot, A.E.I., and Teixeira, M.M. (2011). Therapeutic Effects of Evasin-1, a Chemokine Binding Protein, in Bleomycin-Induced Pulmonary Fibrosis. Am. J. Respir. Cell Mol. Biol. *45*, 72–80. https://doi.org/10.1165/rcmb.2009-0406OC.

51. Keane, M.P., Belperio, J.A., Moore, T.A., Moore, B.B., Arenberg, D.A., Smith, R.E., Burdick, M.D., Kunkel, S.L., and Strieter, R.M. (1999). Neutralization of the CXC chemokine, macrophage inflammatory protein-2, attenuates bleomycin-induced pulmonary fibrosis. J. Immunol. *162*, 5511–5518.

52. Strieter, R.M., Gomperts, B.N., and Keane, M.P. (2007). The role of CXC chemokines in pulmonary fibrosis. J. Clin. Invest. *117*, 549–556. https://doi.org/10.1172/JCI30562.

53. Sung, E.-A., Park, M.H., Henegariu, O., Sime, P.J., and Chae, W.-J. (2023). Dickkopf1 Promotes Pulmonary Fibrosis upon Bleomycin-Induced Lung Injury. Am. J. Pathol. *193*, 1130–1142. https://doi.org/10.1016/j.ajpath.2023.05.009.

54. Yang, P., Luo, Q., Wang, X., Fang, Q., Fu, Z., Li, J., Lai, Y., Chen, X., Xu, X., Peng, X., et al. (2023). Comprehensive Analysis of Fibroblast Activation Protein Expression in Interstitial Lung Diseases. Am. J. Respir. Crit. Care Med. *207*, 160–172. https://doi.org/10.1164/rccm.202110-2414OC.

55. Matute-Bello, G., Wurfel, M.M., Lee, J.S., Park, D.R., Frevert, C.W., Madtes, D.K., Shapiro, S.D., and Martin, T.R. (2007). Essential Role of MMP-12 in Fas-Induced Lung Fibrosis. Am. J. Respir. Cell Mol. Biol. *37*, 210–221. https://doi.org/10.1165/rcmb.2006-0471OC.

56. Dancer, R.C.A., Wood, A.M., and Thickett, D.R. (2011). Metalloproteinases in idiopathic pulmonary fibrosis. Eur. Respir. J. *38*, 1461–1467. https://doi.org/10.1183/09031936.00024711.

57. Senoo, T., Hattori, N., Tanimoto, T., Furonaka, M., Ishikawa, N., Fujitaka, K., Haruta, Y., Murai, H., Yokoyama, A., and Kohno, N. (2010). Suppression of plasminogen activator inhibitor-1 by RNA interference attenuates pulmonary fibrosis. Thorax *65*, 334–340. https://doi.org/10.1136/thx.2009.119974.

58. Oldham, J.M., Huang, Y., Bose, S., Ma, S.-F., Kim, J.S., Schwab, A., Ting, C., Mou, K., Lee, C.T., Adegunsoye, A., et al. (2024). Proteomic Biomarkers of Survival in Idiopathic Pulmonary Fibrosis. Am. J. Respir. Crit. Care Med. *209*, 1111–1120. https://doi.org/10.1164/rccm.202301-0117OC.

59. Herrera, J.A., Dingle, L., Montero, M.A., Venkateswaran, R.V., Blaikley, J.F., Lawless, C., and Schwartz, M.A. (2022). The UIP/IPF fibroblastic focus is a collagen biosynthesis factory embedded in a distinct extracellular matrix. JCI Insight *7*, e156115. https://doi.org/10.1172/jci.insight.156115.

60. Naik, P.K., Bozyk, P.D., Bentley, J.K., Popova, A.P., Birch, C.M., Wilke, C.A., Fry, C.D., White, E.S., Sisson, T.H., Tayob, N., et al. (2012). Periostin promotes fibrosis and predicts progression in patients with idiopathic

CellPress
OPEN ACCESS

pulmonary fibrosis. Am. J. Physiol. Lung Cell Mol. Physiol. *303*, L1046–L1056. https://doi.org/10.1152/ajplung.00139.2012.

61. Vuga, L.J., Milosevic, J., Pandit, K., Ben-Yehudah, A., Chu, Y., Richards, T., Sciurba, J., Myerburg, M., Zhang, Y., Parwani, A.V., et al. (2013). Cartilage Oligomeric Matrix Protein in Idiopathic Pulmonary Fibrosis. PLoS One *8*, e83120. https://doi.org/10.1371/journal.pone.0083120.
62. Booth, A.J., Hadley, R., Cornett, A.M., Dreffs, A.A., Matthes, S.A., Tsui, J.L., Weiss, K., Horowitz, J.C., Fiore, V.F., Barker, T.H., et al. (2012). Acellular Normal and Fibrotic Human Lung Matrices as a Culture System for In Vitro Investigation. Am. J. Respir. Crit. Care Med. *186*, 866–876. https://doi.org/10.1164/rccm.201204-0754OC.
63. Aumiller, V., Strobel, B., Romeike, M., Schuler, M., Stierstorfer, B.E., and Kreuz, S. (2017). Comparative analysis of lysyl oxidase (like) family members in pulmonary fibrosis. Sci. Rep. *7*, 149. https://doi.org/10.1038/s41598-017-00270-0.
64. Chang, J., Lucas, M.C., Leonte, L.E., Garcia-Montolio, M., Singh, L.B., Findlay, A.D., Deodhar, M., Foot, J.S., Jarolimek, W., Timpson, P., et al. (2017). Pre-clinical evaluation of small molecule LOXL2 inhibitors in breast cancer. Oncotarget *8*, 26066–26078. https://doi.org/10.18632/oncotarget.15257.
65. Findlay, A., Turner, C., Schilter, H., Deodhar, M., Zhou, W., Perryman, L., Foot, J., Zahoor, A., Yao, Y., Hamilton, R., et al. (2021). An activity-based bioprobe differentiates a novel small molecule inhibitor from a LOXL2 antibody and provides renewed promise for anti-fibrotic therapeutic strategies. Clin. Transl. Med. *11*, e572. https://doi.org/10.1002/ctm2.572.
66. Wu, S., Zheng, Q., Xing, X., Dong, Y., Wang, Y., You, Y., Chen, R., Hu, C., Chen, J., Gao, D., et al. (2018). Matrix stiffness-upregulated LOXL2 promotes fibronectin production, MMP9 and CXCL12 expression and BMDCs recruitment to assist pre-metastatic niche formation. J. Exp. Clin. Cancer Res. *37*, 99. https://doi.org/10.1186/s13046-018-0761-z.
67. Peng, T., Deng, X., Tian, F., Li, Z., Jiang, P., Zhao, X., Chen, G., Chen, Y., Zheng, P., Li, D., and Wang, S. (2019). The interaction of LOXL2 with GATA6 induces VEGFA expression and angiogenesis in cholangiocarcinoma. Int. J. Oncol. *55*, 657–670. https://doi.org/10.3892/ijo.2019.4837.
68. Umana-Diaz, C., Pichol-Thievend, C., Marchand, M.F., Atlas, Y., Salza, R., Malbouyres, M., Barret, A., Teillon, J., Ardidie-Robouant, C., Ruggiero, F., et al. (2020). Scavenger Receptor Cysteine-Rich domains of Lysyl Oxidase-Like2 regulate endothelial ECM and angiogenesis through non-catalytic scaffolding mechanisms. Matrix Biol. *88*, 33–52. https://doi.org/10.1016/j.matbio.2019.11.003.
69. Zaffryar-Eilot, S., Marshall, D., Voloshin, T., Bar-Zion, A., Spangler, R., Kessler, O., Ghermazien, H., Brekhman, V., Suss-Toby, E., Adam, D., et al. (2013). Lysyl oxidase-like-2 promotes tumour angiogenesis and is a potential therapeutic target in angiogenic tumours. Carcinogenesis *34*, 2370–2379. https://doi.org/10.1093/carcin/bgt241.
70. Puxeddu, I., Pang, Y.Y., Harvey, A., Haitchi, H.M., Nicholas, B., Yoshisue, H., Ribatti, D., Clough, G., Powell, R.M., Murphy, G., et al. (2008). The soluble form of a disintegrin and metalloprotease 33 promotes angiogenesis: Implications for airway remodeling in asthma. J. Allergy Clin. Immunol. *121*, 1400–1406.e14064. https://doi.org/10.1016/j.jaci.2008.03.003.
71. Reichmann, M.T., Tezera, L.B., Vallejo, A.F., Vukmirovic, M., Xiao, R., Reynolds, J., Jogai, S., Wilson, S., Marshall, B., Jones, M.G., et al. (2021). Integrated transcriptomic analysis of human tuberculosis granulomas and a biomimetic model identifies therapeutic targets. J. Clin. Invest. *131*, e148136. https://doi.org/10.1172/JCI148136.
72. Tekin, H., Simmons, S., Cummings, B., Gao, L., Adiconis, X., Hession, C.C., Ghoshal, A., Dionne, D., Choudhury, S.R., Yesilyurt, V., et al. (2018). Effects of 3D culturing conditions on the transcriptomic profile of stem-cell-derived neurons. Nat. Biomed. Eng. *2*, 540–554. https://doi.org/10.1038/s41551-018-0219-9.
73. Schwartz, M.A., and Chen, C.S. (2013). Deconstructing Dimensionality. Science *339*, 402–404. https://doi.org/10.1126/science.1233814.
74. Junqueira, L.C., Bignolas, G., and Brentani, R.R. (1979). Picrosirius staining plus polarization microscopy, a specific method for collagen detection in tissue sections. Histochem. J. *11*, 447–455. https://doi.org/10.1007/BF01002772.
75. Lattouf, R., Younes, R., Lutomski, D., Naaman, N., Godeau, G., Senni, K., and Changotade, S. (2014). Picrosirius Red Staining: A Useful Tool to Appraise Collagen Networks in Normal and Pathological Tissues. J. Histochem. Cytochem. *62*, 751–758. https://doi.org/10.1369/0022155414545787.
76. James, D.S., Brereton, C.J., Davies, D.E., Jones, M.G., and Campagnola, P.J. (2021). Examining lysyl oxidase-like modulation of collagen architecture in 3D spheroid models of idiopathic pulmonary fibrosis via second-harmonic generation microscopy. J. Biomed. Opt. *26*, 066501. https://doi.org/10.1117/1.JBO.26.6.066501.
77. Sarrias, M.R., Gronlund, J., Padilla, O., Madsen, J., Holmskov, U., and Lozano, F. (2004). The Scavenger Receptor Cysteine-Rich (SRCR) Domain: An Ancient and Highly Conserved Protein Module of the Innate Immune System. CRI *24*. https://doi.org/10.1615/CritRevImmunol.v24.i1.10.
78. Lugassy, J., Zaffryar-Eilot, S., Soueid, S., Mordoviz, A., Smith, V., Kessler, O., and Neufeld, G. (2012). The Enzymatic Activity of Lysyl Oxidas-like-2 (LOXL2) Is Not Required for LOXL2-induced Inhibition of Keratinocyte Differentiation. J. Biol. Chem. *287*, 3541–3549. https://doi.org/10.1074/jbc.M111.261016.
79. Soneson, C., Love, M.I., and Robinson, M.D. (2016). Differential analyses for RNA-seq: transcript-level estimates improve gene-level inferences. F1000Res *4*, 1521. https://doi.org/10.12688/f1000research.7563.1.
80. Robinson, M.D., McCarthy, D.J., and Smyth, G.K. (2010). edgeR: a Bioconductor package for differential expression analysis of digital gene expression data. Bioinformatics *26*, 139–140. https://doi.org/10.1093/bioinformatics/btp616.
81. Hänzelmann, S., Castelo, R., and Guinney, J. (2013). GSVA: gene set variation analysis for microarray and RNA-Seq data. BMC Bioinf. *14*. https://doi.org/10.1186/1471-2105-14-7.
82. Ritchie, M.E., Phipson, B., Wu, D., Hu, Y., Law, C.W., Shi, W., and Smyth, G.K. (2015). limma powers differential expression analyses for RNA-sequencing and microarray studies. Nucleic Acids Res. *43*, e47. https://doi.org/10.1093/nar/gkv007.
83. Naba, A., Clauser, K.R., Ding, H., Whittaker, C.A., Carr, S.A., and Hynes, R.O. (2016). The extracellular matrix: Tools and insights for the "omics" era. Matrix Biol. *49*, 10–24. https://doi.org/10.1016/j.matbio.2015.06.003.
84. Szklarczyk, D., Morris, J.H., Cook, H., Kuhn, M., Wyder, S., Simonovic, M., Santos, A., Doncheva, N.T., Roth, A., Bork, P., et al. (2017). The STRING database in 2017: quality-controlled protein–protein association networks, made broadly accessible. Nucleic Acids Res. *45*, D362–D368. https://doi.org/10.1093/nar/gkw937.
85. Yao, L., Conforti, F., Hill, C., Bell, J., Drawater, L., Li, J., Liu, D., Xiong, H., Alzetani, A., Chee, S.J., et al. (2019). Paracrine signalling during ZEB1-mediated epithelial–mesenchymal transition augments local myofibroblast differentiation in lung fibrosis. Cell Death Differ. *26*, 943–957. https://doi.org/10.1038/s41418-018-0175-7.
86. Russell, H.K. (1972). A modification of Movat's pentachrome stain. Arch. Pathol. *94*, 187–191.

STAR★METHODS

KEY RESOURCES TABLE

REAGENT or RESOURCE	SOURCE	IDENTIFIER
**Antibodies**		
AB0023	Gilead Sciences, Inc	N/A
Isotype Control	Gilead Sciences, Inc	N/A
Rat Anti-Human Procollagen Type 1 Monoclonal Antibody	Abcam plc.	RRID:AB_1142324
Tenascin C antibody	Abcam plc.	RRID:AB_10865908
**Biological samples**		
IPF and normal samples as reported in experimental model and subject details	University Hospital Southampton	N/A
**Chemicals, peptides, and recombinant proteins**		
PXS-S2A	Pharmaxis Ltd.	N/A
Penicillin/Streptomycin	Sigma-Aldrich	Cat#P4333
DMEM	Thermo-Fisher Scientific	Cat#11965092
F12	Thermo-Fisher Scientific	Cat#11765054
Human large vessel endothelial cell basal media	Thermo-Fisher Scientific	Cat#M200500
Geltrex	Thermo-Fisher Scientific	Cat#A1413202
Low serum Growth Supplement	Thermo-Fisher Scientific	Cat#S00310
Fetal Bovine Serum	Thermo-Fisher Scientific	Cat#10500-064
NEAA	Thermo-Fisher Scientific	Cat#11140-035
Sodium pyruvate	Thermo-Fisher Scientific	Cat#11360-039
L-glutamine	Sigma-Aldrich	Cat#59202C
Amphotericin B	Thermo-Fisher Scientific	Cat#15290-026
Insulin	Sigma-Aldrich	Cat#I9278-5ML
Adenine	Sigma-Aldrich	Cat#A2786-5G
Hydrocortisone	Sigma-Aldrich	Cat#H0888-1G
Epidermal Growth Factor	R&D Systems	Cat#236-EG-200
L-Ascorbic Acid	Sigma-Aldrich	Cat#A8960-5G
Trypsin-EDTA	Thermo-Fisher Scientific	Cat#25300054
Strataclean resin	Agilent	Cat#400714
Calcein AM	Thermo-Fisher Scientific	Cat#C3099
Trypsin Neutraliser Solution	Thermo-Fisher Scientific	Cat# R002100
**Critical commercial assays**		
Monarch Total RNA Miniprep kit	New England Biolabs	Cat#T2010S
Human Luminex Discovery Assay Targeting GDF15, TIMP1, TNC, Periostin, OPN, VEGFA, MCP1,CCL4, CCL3, CXCL2, DKK1, FAP, MMP12, PAI1	R&D systems	Cat#LXSAHM
RNAscope 2.5 HD Duplex Assay using probe Probe-Hs-LOXL2-C1, Probe-Hs-PLOD2-C2,	ACD Bio	Cat#322435<br>Cat#311341<br>Cat#547761-C2
Human VEGF DuoSet ELISA	R&D Systems	Cat#DY293B-05
Human Fibronectin DuoSet ELISA	R&D Systems	Cat#DY1918-05
TaqMan Fast Advanced qPCR using primers targeting *COL3A1, PLOD2, P4HA3* and *TWIST1*	Thermo Fisher Scientific	Cat#4444963<br>Cat#4331182
**Deposited data**		
Spatial transcriptome profiling identifies CREB1 as a regulator of core transcriptional programs in idiopathic pulmonary fibrosis	NCBI Gene Expression omnibus	GSE169500

(*Continued on next page*)

Continued

REAGENT or RESOURCE	SOURCE	IDENTIFIER
Spatially resolved deconvolution of the fibrotic niche in lung fibrosis	Cell Reports, Table S1 Eyres et al.[36]	https://doi.org/10.1016/j.celrep.2022.111230
Experimental models: Cell lines		
Human umbilical vascular endothelial cells (HUVECs)	Sigma-Aldrich	Cat#C-12200
Parenchymal Lung fibroblasts as reported in experimental model and subject details	University Hospital Southampton	N/A
Software and algorithms		
R	The R Software Foundation	Version 4.2.2
GraphPad prism	GraphPad	Version 10.0.2
LAS X	Leica Microsystems	Version 3.7.4
SquisherJoy software	CellScale	Version 5.23
Protein Lynx Global Server (PLGS)	Waters	Version 3.0
Bio-Rad CFX Maestro	Bio-Rad	Version 2.3
WimTube	Wimasis	Version 1.0

RESOURCE AVAILABILITY

Lead contact

Further information and requests for resources and reagents should be directed to and will be fulfilled by the lead contact, Professor Mark Jones, mark.jones@soton.ac.uk.

Materials availability

This study did not generate new unique reagents.

Data and code availability

- RNAseq data from the cell culture studies can be accessed via NCBI Gene Expression Omnibus (GEO) ID GSE255705 (https://www.ncbi.nlm.nih.gov/geo/query/acc.cgi?acc=GSE255705). All other data generated during this study are included in the manuscript and supporting files. This paper also analyses existing, publicly available data. The spatial transcriptomic laser capture microdissection dataset of IPF and control lung tissue is available via NCBI Gene Expression Omnibus ID GSE169500. https://www.ncbi.nlm.nih.gov/geo/query/acc.cgi?acc=GSE169500 and the digital spatial profiling dataset of IPF and control lung tissue is available within the supplementary dataset of publication Cell Reports. 40:111230. https://doi.org/10.1016/j.celrep.2022.111230.
- All original code is available in this paper's supplemental information in Data S3.
- Any additional information required to reanalyze the data reported in this paper is available from the lead contact upon request.

EXPERIMENTAL MODEL AND SUBJECT DETAILS

Clinical samples

Studies were approved by the Southampton and South West Hampshire and the Mid and South Buckinghamshire Local Research Ethics Committees (ref. 07/H0607/73, 14/SC/0186 & 348/03/T), and all subjects gave written informed consent. Clinically indicated IPF lung biopsy tissue samples deemed surplus to clinical diagnostic requirements or non-fibrotic parenchymal lung tissue from patients undergoing surgery for early stage lung cancer (macroscopically normal lung sampled remote from a cancer site) were formalin fixed and paraffin embedded or used to establish parenchymal lung fibroblast cultures.[15] Details of donor characteristics including age, gender, and disease status are provided in Table S1. All IPF samples were from patients subsequently receiving a multidisciplinary diagnosis of IPF according to international consensus guidelines.

Parenchymal lung fibroblast cultures

Primary fibroblast cultures were established from parenchymal lung tissue as previously described.[15] All primary cultures were tested and free of mycoplasma contamination. Sample sizes were selected based on previous comparable studies in the labs in which these studies were performed.[15,22,37]

2D cell culture,[37] Scar-in-A-Jar[38] and the 3D spheroid model[15,22] culture systems used the standard media condition and time for each model system, and a second timepoint (2 weeks) to allow for overlap of culture duration. For this later timepoint, the serum concentration was reduced to 0.5% in the 2D cell culture and Scar-In-A-Jar models to reduce the likelihood of overgrowth of the cell

monolayers. The 3D spheroid culture was performed as previously described,[15] sampling at 2 weeks, as well as the standard 6-week timepoint. For each standard condition and time, cells were cultured in the absence or presence of TGF-β1 (3 ng/mL).

Primary human umbilical vein endothelial cells (HUVEC)

HUVECs were purchased from ThermoFisher Scientific (Cat#C0035C) and were human umbilical vein cells from a single, newborn (<14days), female donor. They were cultured on GelTrex basement membrane extract coated plates in human large vessel endothelial cell basal media supplemented with low serum growth supplement, before continuing to the tube forming assay (reported in the *In vitro* human umbilical vein endothelial cell assay section of Method Details). They were tested and free of mycoplasma contamination.

METHOD DETAILS

Spatial transcriptomic data analyses

We analyzed transcriptomic datasets that we have recently established. Briefly, the laser capture microdissection dataset (GSE169500)[22] was from Formalin-Fixed Paraffin-Embedded (FFPE) control non-fibrotic lung tissue (alveolar septae, [n = 10]) and usual interstitial pneumonia/idiopathic pulmonary fibrosis FFPE lung tissue (fibroblast foci, [n = 10] and adjacent non-affected alveolar septae, [n = 10]). Fibroblast foci were identified using the ARCTURUS Paradise PLUS FFPE LCM Staining Kit (ThermoFisher Scientific) by the staining pattern of the myxoid extracellular matrix, whilst selection of alveolar septae excluded visible airways and blood vessels. Total RNA was isolated, cDNA libraries were prepared using Ion Ampli-Seq-transcriptome human gene expression kit (Life Technologies, Paisley, UK) and sequenced using Ion Torrent Proton Sequencer. A two-stage mapping strategy was used to map the reads to UCSC hg19 human genome. Raw counts data were then normalised to counts per million mapped reads using edgeR.

The digital spatial profiling dataset[36] for LOXL2 spatial expression we previously generated from Nanostring GeoMx CTA profiling of 1813 unique genes within 60 regions of interest (control alveolar septae, IPF distant alveolar septae, IPF adjacent alveolar septae, IPF fibroblastic foci, IPF immune infiltrates, IPF blood vessels, and control blood vessels). Full data are available within the published supplemental files.[36]

Carmine red staining of fixed spheroids

To assess the 3D morphology of spheroids, the multispectral autofluorescence of carmine red and confocal microscopy were used. Spheroids were fixed in PFA and stored in PBS before post-fixing in 48% ethanol:2% glacial acetic acid: 10% formaldehyde and staining in Langeron's carmine. Following differentiation in acid alcohol, stained spheroids were optically-cleared by dehydration through a graded methanol series (50%, 70%, 80%, 95%, 100%, 2 changes of each), then 2:1 dichloromethane (DCM):methanol, followed by 2 changes of DCM and 3 changes of dibenzylether (DBE). Samples were imaged in DBE in glass bottom Ibidi chamber-slides on a Leica TCS-SP8 confocal microscope on an inverted dmi8 microscope stand using an HC PL APO CS2 20x/0.75 IMM objective with glycerol:water (8:2) as immersion fluid. Multichannel imaging was undertaken using system-optimised Z spacing, excitation at 488 and 561nm and capturing carmine autofluorescence emission at bandwidths of 500–550 and 690-740nm (green, red false colors respectively).

RNAseq and bioinformatic analyses

Cells were harvested at specified timepoints for each model system and RNA isolated using the RNeasy Mini kits (Qiagen, Manchester, UK) according to the manufacturer's instructions. For the 3D spheroid model, RLT buffer also contained Proteinase K (Sigma, Poole, UK) to aid digestion of the ECM and facilitate cell lysis.

RNAseq was performed by Novogene (UK) using an Illumina Novaseq 6000 sequencer. cDNA libraries were mRNA enriched using polyA enrichment, and ~150bp paired-end reads were quantified from these cDNA libraries. Raw fastq files were pseudoaligned to human reference transcriptome hg38, derived from human reference genome GRCh38, using RefSeq's transcripts to construct an index file. Pseudoalignments were completed using Kallisto running in paired end mode, to calculate raw counts.

Raw, transcript-level counts data were imported into R using the tximport package or downloaded from the Gene Expression Omnibus.[79] Differential expression analysis was performed using edgeR[80] with multiple test correction performed using the Benjamini-Hochberg methodology. Counts per million reads were calculated using edgeR for models and laser capture microdissection data. LCMD and models data were combined before Gene Set Variation Analysis was performed using the gsva R package, with differential scores calculated between all groups using a Kruskal Wallis test with B-H multiple test correction. with differential regulation of gene sets assessed using the Limma R package.[81,82] Graphs were produced using ggplot2.

Proteomic analysis

Proteomic analysis was performed on 3D spheroids and their secreted proteins; for secretome studies, spheroids were washed and cultured in serum free media for 24 h prior to collection of the conditioned medium for analysis.

Spheroids were pooled in pairs to yield approximately 100 μg protein per sample and were lysed in 0.1% sodium dodecyl sulfate in an ultrasonic bath followed by a sonication probe.

Protein lysates were precipitated by methanol/chloroform extraction then reduced for 1 h with 1 mM dithiothreitol followed by alkylation for 45 min with 5.5 mM iodoacetamide in the dark at room temperature. Samples were digested with endoproteinase Lys-C for 4 h then 2 μg trypsin overnight at room temperature. Enolase and ClpB internal reference standards were spiked at 300 fmol. Peptides were then fractionated by OFFgel electrophoresis into 12 peptide fractions according to manufacturer's instructions. Each fraction was purified using a C18 Empore 96-well solid phase extraction plate, before evaporation to dryness and resuspension in loading buffer (3% acetonitrile +0.1% formic acid) for mass spectrometry analysis. Each experiment was performed in triplicate. For the secretome, media from five individual culture wells per cell donor were combined. Protein was bound to StrataClean resin beads (Agilent) by incubation for 1 h at 2-8°C, followed by sequential reduction using DTT, alkylation using iodoacetamide, and digestion with sequencing grade trypsin. After elution from the beads, samples were subjected to SPE clean-up on C18 columns and sample concentration.

Fractions were analyzed by UPLC-HDMS^E. Half of each fraction was injected and peptides were separated by liquid chromatography using a NanoAcquity UPLC system (Waters, Elstree, UK) with a C18 reverse-phase column at a flow rate of 300 nL/min over a 3–50% 80% acetonitrile/dH_2O + 0.1% formic acid gradient of 90 min. Peptide ions were sprayed into a Waters Synapt G2-S system operating in positive ion mode, with ion mobility enabled prior to fragmentation. Data were collected in MSE mode of acquisition, alternating between low energy (5V) and high energy (15V–45V ramp) scans. Glu-fibrinopeptide (m/z = 785.8426, 100 fmol/μL) was used as LockMass and was sampled every 60 s for calibration.

Raw data files were processed using Protein Lynx Global Server (PLGS) version 3.0 (Waters, Elstree, UK). Data were searched against the human UniProt database (downloaded 29/11/2013) using an Ion Accounting algorithm in PLGS 3.0.2.

Proteomic data were processed in R, with proteins identified in at least one sample in the fibroblastic foci proteomic data[59] and the 3D model proteomic data included. Extracellular matrix proteins including core matrix proteins, secreted proteins and basement membrane proteins were acquired from Naba et al.[83] Basement membrane proteins were excluded from both the fibroblastic focus and 3D spheroid model proteomes, before cross-referencing protein names to identify common and different ECM proteins between the two datasets. IPF extracellular proteomic data were derived from Booth et al.,[62] and the most abundant extracellular matrix proteins present in decellularized IPF tissue excluding basement membrane proteins, ordered by their abundance in the Booth dataset were cross-referenced with the 3D spheroid model proteome and the fibroblastic focus proteome.

3D proteomic data was also cross-referenced with proteins identified as having a hazard ration >1 in a study of proteomic biomarkers of outcome in IPF.[58] Protein lists were mapped along with up to 50 primary and secondary interaction partners using STRING (v11)[84] to demonstrate their interaction network (Figure S2).

Luminex analysis

Supernatants derived from each model system were taken and analyte concentration in the supernatant was quantified against a standard curve using a Human Luminex Discovery Assay Kit (R&D systems LXSAHM), analyzed on the Magpix Luminex platform. Luminex data were normalised to total RNA and media volume to allow comparison across different culture models.

Immunohistochemistry

Sections (4 μm) of lung tissue or 3D spheroid model (fixed in 4% paraformaldehyde and paraffin embedded) were processed and stained as previously described.[15,37,85] Briefly, the tissue sections were de-waxed, rehydrated and incubated with 3% hydrogen peroxide in methanol for 10 min to block endogenous peroxidase activity. Sections were then blocked with normal goat serum and incubated at room temperature with a primary antibody against tenascin C (1:500, Abcam ab108930) or procollagen type I (1:100, Abcam ab64409); after washing, bound antibody was detected using a biotinylated secondary antibody (1:500, Vector Laboratories Ltd., UK) followed by streptavidin-conjugated horseradish peroxidase and visualisation using DAB. Sections were counterstaining with Gill's Haematoxylin. For co-localisation studies, adjacent serial sections were stained using modified Movat's Pentachrome Stain.[86] Images were acquired using an Olympus Dotslide Scanner VS110.

Transmission electron microscopy (TEM)

TEM was performed on 3D spheroids as previously described.[15] Briefly, spheroids were fixed in 3% glutaraldehyde in 0.1 M cacodylate buffer at pH 7.4 for electron microscopy. Samples were then post-fixed sequentially in osmium/ferrocyanide fixative, thiocarbohydrazide solution, osmium tetroxide, uranyl acetate and Walton's lead aspartate solution before dehydration in graded ethanol and acetonitrile. Samples were embedded in Spurr resin and 100 nm ultra-thin sections visualised using an FEI Tecnai 12 transmission electron microscope (FEI Company, Hillsboro, OR, USA).

LOXL2 serum assay

LOXL2 concentration was measured in serum as previously reported.[65] Briefly, a sandwich using two commercially available antibodies and a high sensitivity detection method were used to determine LOXL2 concentrations. Calibrations curves were derived from testing human recombinant LOXL2.

RNA in situ hybridisation

Simultaneous *in situ* detection of the LOXL2 and PLOD2 mRNA on formalin-fixed paraffin-embedded tissue sections was performed using duplex RNAscope technology (Advanced Cell Diagnostics, Biotechne, Abingdon, UK) as previously reported.[22] Briefly, LOXL2 was detected by C1-probe (Probe-Hs-LOXL2-C1, 311341) and PLOD2 was detected by C2-probe (Probe-Hs-PLOD2-C2, 547761-C2). 5 μm sections were baked at 60°C, deparaffinised in xylene, followed by rehydration in an ethanol series. Target retrieval, hybridisation with target probes, amplification, and chromogenic detection were performed according to the manufacturer's recommendations (RNAscope 2.5 Duplex Detection protocol for FFPE tissues). Sections were counterstained with Gill's Hematoxylin, and mounted with Vectamount permanent mounting medium prior to imaging. Assays were performed with duplex positive (PPIB and POLR2A) and negative controls. Images were acquired using an Olympus Dotslide Scanner VS110 (Olympus UK, Southend-on-Sea, UK).

Evaluation of AB0023 in the 3D spheroid model

For studies of AB0023 in the 3D spheroid model, all experiments were performed in the presence of TGF-β1 (3 ng/mL), the condition that most closely recapitulated a fibroblastic focus. AB0023 or an isotype control IgG antibody were tested at 2.5 μg/ml and 25 μg/ml and were present in the medium for the duration of the culture and were replenished every 3 days. As a positive control, we also included PXS-S2A at 100nM, a concentration which we have shown to inhibit LOXL2[15] and its vehicle control (0.1%DMSO). AB0023 and the isotype control IgG antibody were kindly supplied by Gilead, while PXS-S2A was from Pharmaxis. After 6 weeks of culture, the cell-free medium was harvested for measurement of LOXL2 activity and the spheroids processed for analysis of tissue stiffness, total protein, collagen and pyridinoline cross-links, all as detailed below.

LOXL2 activity assay

LOXL2 catalytic amine oxidase activity within the cell conditioned media of 3D spheroids was measured as previously reported using the PXS-5878/Simoa platform.[65] Briefly, an activity-based probe, PXS-5878 which potently and irreversibly binds to unoccupied lysyl oxidase active sites was used in combination with a LOXL2 capturing antibody.

Protein, hydroxyproline and collagen cross-link analysis

Analysis was performed as previously described.[15,22] Briefly, samples were thawed and reduced with KBH4 before acid hydrolysis in 6M HCl at 102°C for 18 h. Cooled hydrolyzed samples were evaporated to dryness under vacuum and then resuspended in 200 μL HPLC-grade H2O. Total protein was quantified in the hydrolyzed samples using a genipin-based amino acid assay (QuickZyme Biosciences, Leiden, The Netherlands), using standard hydrolyzed bovine serum albumin as standard. Total collagen content was estimated using colorimetric assay of hydroxyproline (Hyp) based on the reaction of oxidized hydroxyproline with 4-(Dimethylamino) benzaldehyde, as per manufacturer's instruction (Sigma-Aldrich, Poole, UK). The molar content of collagen was estimated from hydroxyproline using a conversion factor of 300 hydroxyprolines per triple helix, and mass of collagen was estimated using a molecular weight of 300 kDa per triple helix. Total mature pyridinium cross-links (PYD +DPD) were determined using enzyme-linked immunosorbent assay (ELISA; Quidel Corporation, San Diego, USA) according to manufacturer's instructions. Quantitation of the collagen cross-links and total collagen was achieved by comparing to a standard curve. Sample values were interpolated using GraphPad Prism seven software.

Parallel plate compression testing

Analysis was performed as previously described.[15] Briefly, to determine the stiffness characteristics (Young's modulus, E) of the 3D *in vitro* model of fibrosis, cultures were subjected to parallel plate compression testing using a CellScale MicroSquisher fitted with a round tungsten cantilever (thickness 406.4 nm) and accompanying SquisherJoy V5.23 software (CellScale, Ontario, Canada). Analysis of stress vs. strain relationships was carried out using the compression phase of the fifth cycle from where sample stiffness can be inferred. Force and displacement data were transformed to engineering stress versus engineering strain plots using the horizontal cross-sectional diameter of the sample immediately before the start of each test. Young's modulus (E), a measure of stiffness, was calculated using a modified Hertzian half-space contact mechanics model for elastic spheres as previously described.[15,22]

Enzyme-linked immuno-sorbent assay

VEGFA and Fibronectin concentrations were assayed in conditioned media from 3D spheroid models using Human VEGF DuoSet ELISA and Human Fibronectin DuoSet ELISA kits (RnD systems).

In vitro human umbilical vein endothelial cell assay

Tube formation was performed using human umbilical vein endothelial cells (HUVECs) (supplied by ThermoFisher Scientific) cultured on Geltrex basement membrane extract (also from ThermoFisher Scientific) following the manufacturer's protocol. Briefly, HUVECs were loaded with Calcein AM (Invitrogen) for 30 min before being seeded onto Geltrex-coated 48-well tissue culture plates. Tube formation was induced by addition 2% FBS and bFGF (3 ng/mL) for 16–20 h. AB0023 or isotype control IgG were added at 2.5 or

25 μg/mL. Suramin (30 μM) was included as a positive inhibitor control. Immunofluorescent images of tubes were taken after 24 h and the extent of tube formation assessed by the online image analysis "WimTube tool" (Wimasis, Munich, Germany) to quantify the number of loops, branches and total tube length.

Quantitative real-time PCR (qPCR)

QPCR was performed using TaqMan Fast Advanced Master mix (Thermo Fisher) in a Bio-Rad CFX Opus 96 thermocycler.

QUANTIFICATION AND STATISTICAL ANALYSIS

Statistical analyses were performed in R version 4.2.2, or GraphPad Prism v9.4.1 (GraphPad Software Inc, San Diego, CA) unless otherwise indicated. Details of R analysis can be found in supplemental code (Data S3). No data were excluded from the studies and for all experiments, all attempts at replication were successful. For each experiment, sample size reflects the number of independent biological replicates and is provided in the figure legend. Normality of distribution was assessed using the D'Agostino-Pearson normality test. Statistical analyses of single comparisons of two groups utilised Student's t test. Graphs show the mean and error bars represent standard deviation. For multiple comparisons, one-way analysis of variance (ANOVA) with Šídák's multiple comparisons test or Kruskal-Wallis analysis with Dunn's multiple comparison test were used for parametric and non-parametric data, respectively. Results were considered significant if $p < 0.05$, where *$p < 0.05$, **$p < 0.01$, ***$p < 0.001$, ****$p < 0.0001$. Statistical details can also be found in the figure legends.

Cell Reports Medicine, Volume *5*

Supplemental information

Spatial transcriptomic validation of a biomimetic model of fibrosis enables re-evaluation of a therapeutic antibody targeting LOXL2

Joseph A. Bell, Elizabeth R. Davies, Christopher J. Brereton, Milica Vukmirovic, James J.W. Roberts, Kerry Lunn, Leanne Wickens, Franco Conforti, Robert A. Ridley, Jessica Ceccato, Lucy N. Sayer, David A. Johnston, Andres F. Vallejo, Aiman Alzetani, Sanjay Jogai, Ben G. Marshall, Aurelie Fabre, Luca Richeldi, Phillip D. Monk, Paul Skipp, Naftali Kaminski, Emily Offer, Yihua Wang, Donna E. Davies, and Mark G. Jones

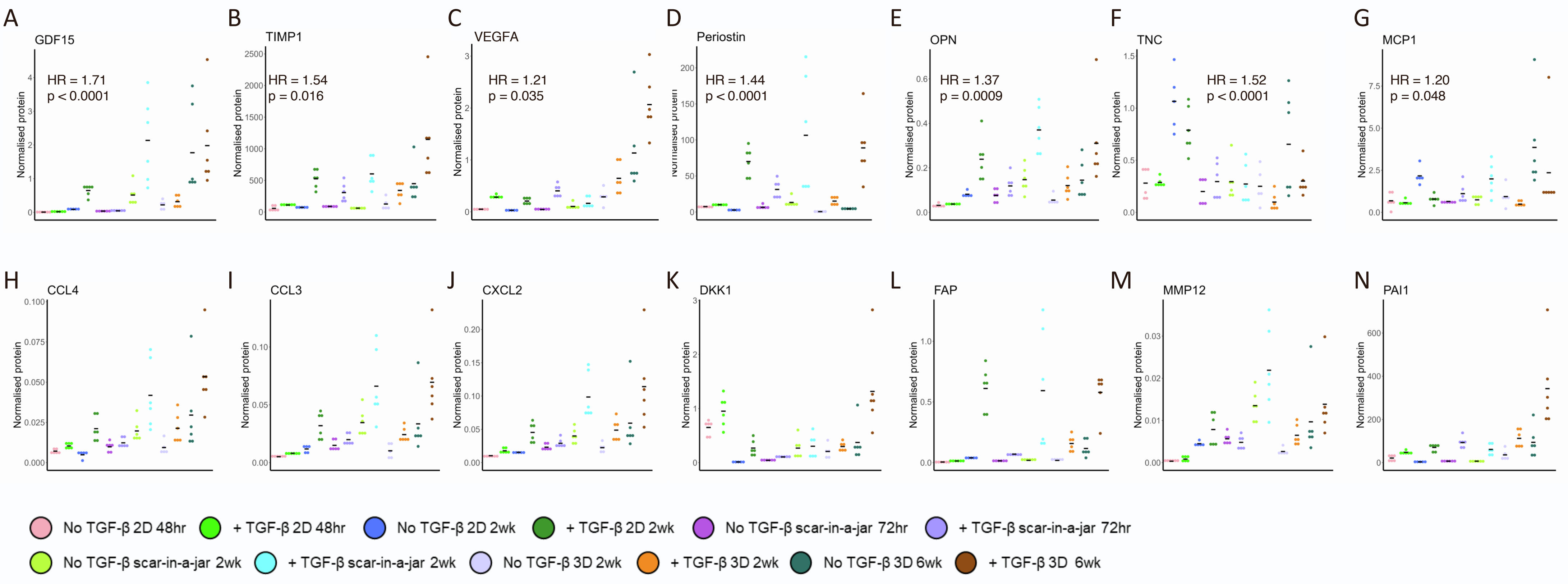

Figure S1. Cytokine and extracellular protein profiles of cell conditioned media from each model system, Related to Figure 3. Individual cytokines and extracellular proteins from conditioned media from each model system. A primary parenchymal lung fibroblast cell line was cultured in standard 2D culture, the Scar-in-a-jar model or the 3D spheroid model in the absence or presence of TGF-β at early and late timepoints (n=6 per condition). (A) GFD15, (B) TIMP1, (C) VEGFA (D) Periostin (E) OPN (osteopontin), (F) TNC), (G), MCP1 (CCL2), (H) CCL4, (I) CCL3, (J) CXCL2, (K) DKK1, (L) FAP, (M) MMP12, (N) PAI1 (A-G) proteins which have a significant hazard ratio > 1 for increased risk of death or transplant in Oldham et al. 2023[58]. Hazard ratios and P values from this study are inset in graphs. Supernatants derived from each model system were taken and analyte concentration in the supernatant was quantified against a standard curve using a Human Luminex Discovery Assay Kit analysed on the Magpix Luminex platform. Luminex data were normalised to total RNA and media volume to allow comparison across different culture models.

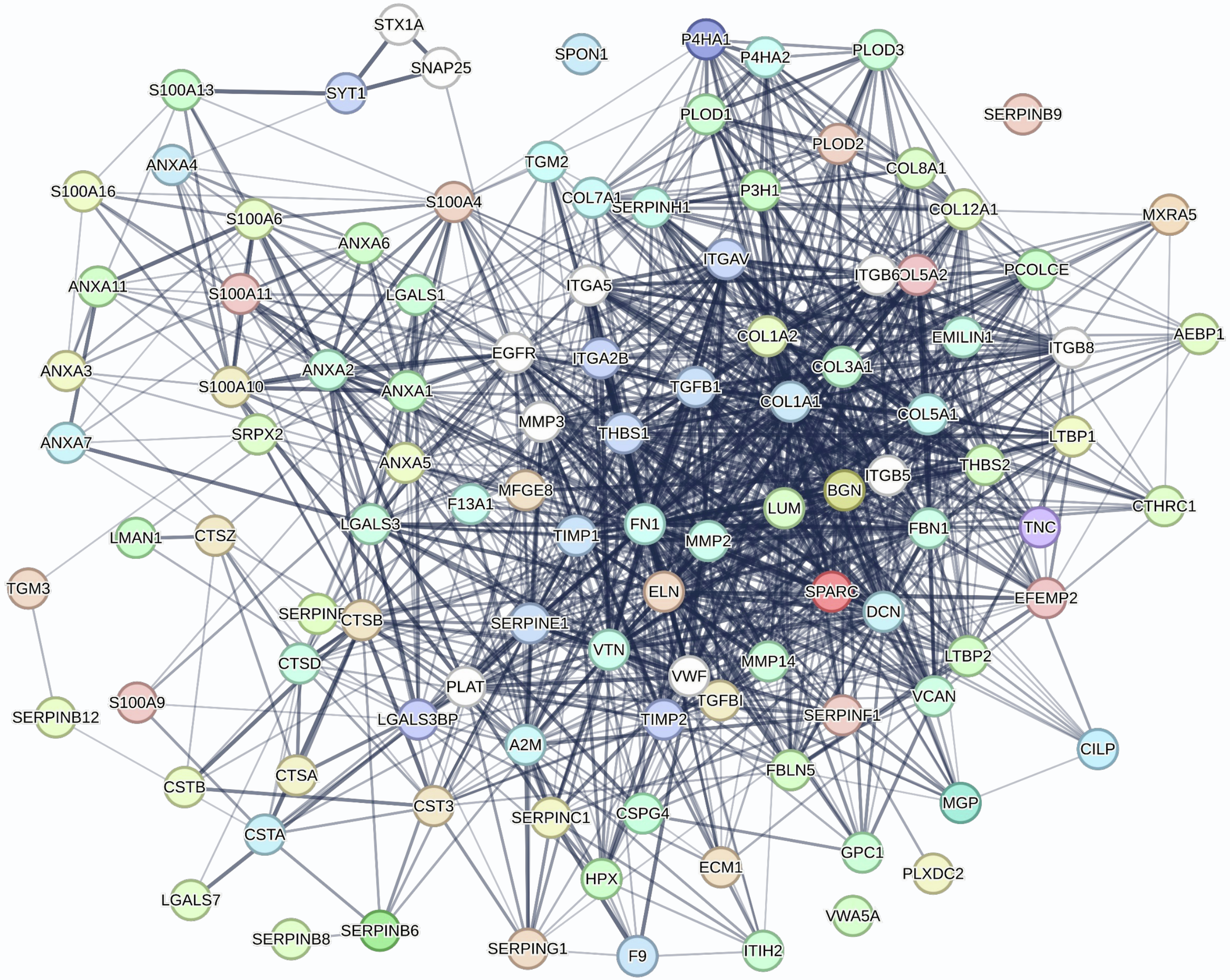

Figure S2. Interaction network map of common proteins between 3D spheroid model and fibroblastic foci, Related to Figure 3. STRING map with up to 10 additional medium-confidence interactors for 88 common extracellular matrix proteins between IPF fibroblastic foci and 3D spheroid model (see Fig. 4a and Supplementary Table 2). Thickness of lines denotes confidence of interaction.

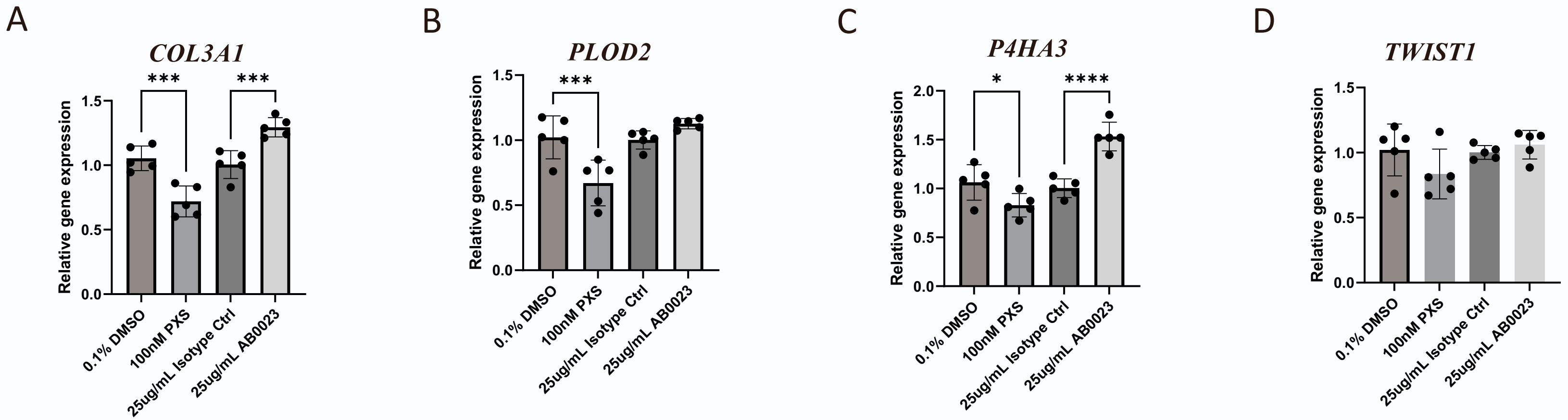

Figure S3. PXS-S2A decreases expression of profibrotic genes, Related to Figure 5. Primary human lung fibroblasts were cultured in 3D spheroid media conditions in the presence of TGF-β and treated with AB0023, an isotype control antibody at the same concentration, PXS-S2A or its vehicle control (0.1% DMSO). Relative gene expression for (A) *COL3A1* (B) *PLOD2* (C) *P4HA3* (D) *TWIST1* determined by qRT-PCR and analyzed using DDCt method (n=5 replicates per condition across 2 independent experiments). *P<0.05, **P<0.01, ***P<0.001, ****P<0.0001. One-way ANOVA with Šídák's multiple comparisons test was used to evaluate statistical significance. (F values: (A) 27.33, (B) 12.12, (C) 23.16, Degrees of Freedom: 16.

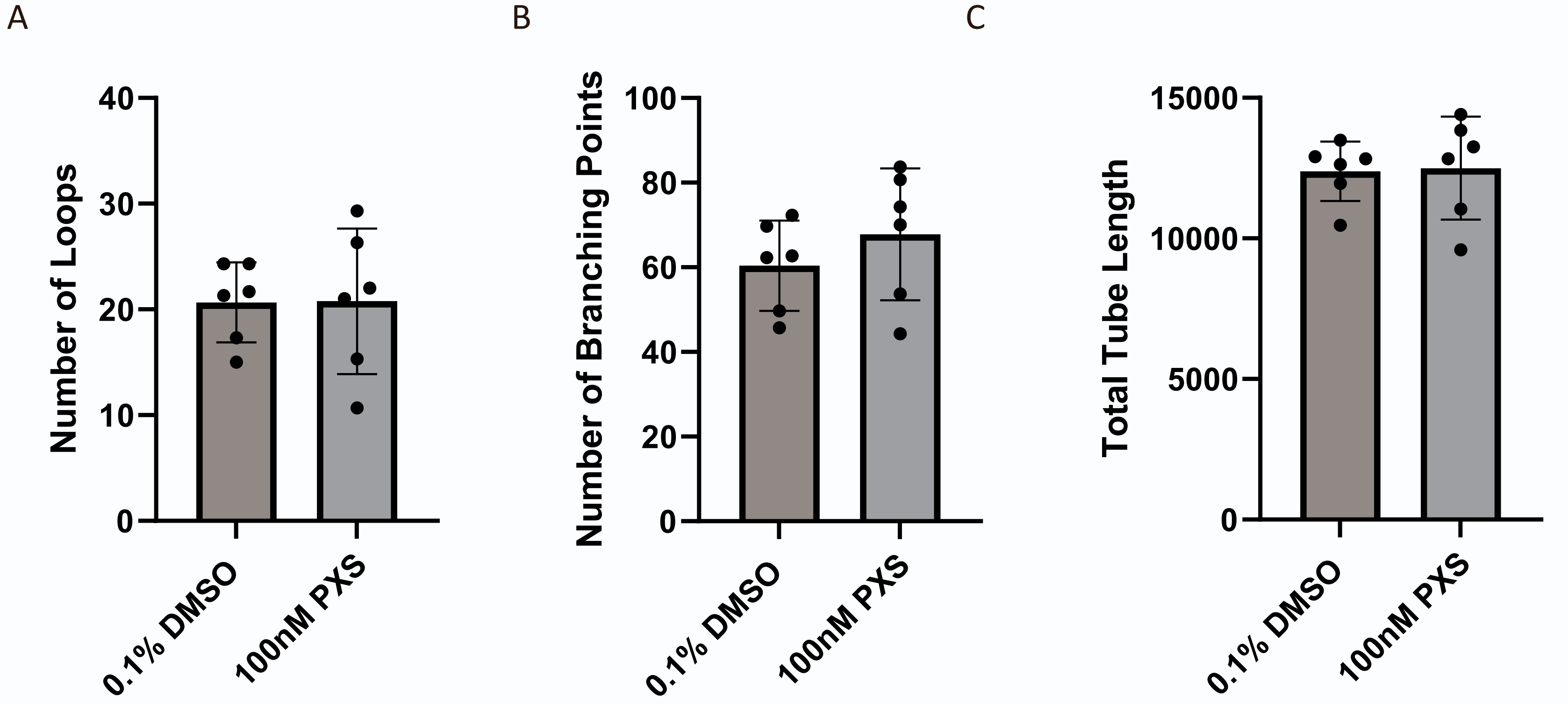

Figure S4. Selective targeting of LOXL2 catalytic activity using the small molecule inhibitor PXS-S2A does not inhibit endothelial tube formation, Related to Figure 6. Endothelial tube formation by human umbilical vein endothelial cells was assessed in the presence of PXS-S2A or 0.1% DMSO (n=6 replicates per condition across 2 independent experiments). Quantification of topological parameters of capillary structure by computer-aided image analysis for (A) number of loops, (B) number of branching points, (C) total tube length. Data are means +/- SD.

Sex	Age	Disease status
M	67	IPF
M	66	IPF
F	63	IPF
M	69	IPF
F	74	IPF
M	69	IPF
F	75	IPF
M	73	IPF
M	71	IPF
M	73	IPF
F	73	Normal
M	68	Normal
M	64	Normal
M	76	Normal
F	61	Normal
M	75	Normal
M	70	Normal
M	72	Normal
F	67	Normal
M	63	Normal

Table S1. Demographic details of donors, related to Figures 1-5.

Supplemental References

S1. Herrera, J.A., Dingle, L., Montero, M.A., Venkateswaran, R.V., Blaikley, J.F., Lawless, C., and Schwartz, M.A. (2022). The UIP/IPF fibroblastic focus is a collagen biosynthesis factory embedded in a distinct extracellular matrix. JCI Insight 7, e156115.

S2. Naba, A., Clauser, K.R., Ding, H., Whittaker, C.A., Carr, S.A., and Hynes, R.O. (2016). The extracellular matrix: Tools and insights for the "omics" era. Matrix Biol. 49, 10–24.

S3. Oldham, J.M., Huang, Y., Bose, S., Ma, S.-F., Kim, J.S., Schwab, A., Ting, C., Mou, K., Lee, C.T., Adegunsoye, A., et al. (2024). Proteomic Biomarkers of Survival in Idiopathic Pulmonary Fibrosis. Am. J. Respir. Crit. Care Med. 209, 1111-1120.
